# Hippo, TGF-β, and Src-MAPK pathways regulate transcription of the *upd3* cytokine in *Drosophila* enterocytes upon bacterial infection

**DOI:** 10.1371/journal.pgen.1007091

**Published:** 2017-11-06

**Authors:** Philip Houtz, Alessandro Bonfini, Xi Liu, Jonathan Revah, Aurélien Guillou, Mickael Poidevin, Korneel Hens, Hsin-Yi Huang, Bart Deplancke, Yu-Chen Tsai, Nicolas Buchon

**Affiliations:** 1 Cornell Institute of Host-Microbe Interactions and Disease. Department of Entomology. Cornell University, Ithaca, NY, United States of America; 2 Institut de Biologie Integrative de la Cellule. Avenue de la Terrasse, France; 3 Centre for Neural Circuits and Behavior, The University of Oxford, Tinsley Building, Mansfield Road, Oxford, United Kingdom; 4 Department of Life Science and Life Science Center, Tunghai University, Taichung, Taiwan, Republic of China; 5 Laboratory of Systems Biology and Genetics (LSBG). School of Life Sciences, École Polytechnique Fédérale de Lausanne, Lausanne, Switzerland; 6 Swiss Institute of Bioinformatics, Lausanne, Switzerland; Seoul National University, REPUBLIC OF KOREA

## Abstract

Cytokine signaling is responsible for coordinating conserved epithelial regeneration and immune responses in the digestive tract. In the *Drosophila* midgut, Upd3 is a major cytokine, which is induced in enterocytes (EC) and enteroblasts (EB) upon oral infection, and initiates intestinal stem cell (ISC) dependent tissue repair. To date, the genetic network directing *upd3* transcription remains largely uncharacterized. Here, we have identified the key infection-responsive enhancers of the *upd3* gene and show that distinct enhancers respond to various stresses. Furthermore, through functional genetic screening, bioinformatic analyses and yeast one-hybrid screening, we determined that the transcription factors Scalloped (Sd), Mothers against dpp (Mad), and D-Fos are principal regulators of *upd3* expression. Our study demonstrates that *upd3* transcription in the gut is regulated by the activation of multiple pathways, including the Hippo, TGF-β/Dpp, and Src, as well as p38-dependent MAPK pathways. Thus, these essential pathways, which are known to control ISC proliferation cell-autonomously, are also activated in ECs to promote tissue turnover the regulation of *upd3* transcription.

## Author summary

Tissue regeneration is a fundamental process that maintains the integrity of the intestinal epithelium when faced with chemical or microbial stresses. In both healthy and diseased conditions, pro-regenerative cytokines function as central coordinators of gut renewal, linking inflammation to stem cell activity. In *Drosophila*, the upstream events that stimulate the production of the primary cytokine Unpaired 3 (Upd3) in response to indigenous or pathogenic microbes have yet to be elucidated. In this study, we demonstrate that *upd3* expression is driven in different cell types by separate microbe-responsive enhancers. In enterocytes (ECs), cytokine induction relies on the Yki/Sd, Mad/Med, and AP-1 transcription factors (TFs). These TF complexes are activated downstream of the Hippo, TGF-β and Src-MAPK pathways, respectively. Inhibiting these pathways in ECs impairs *upd3* transcription, which in turn blocks intestinal stem cell proliferation and reduces the survival rate of adult flies following enteric infections. Altogether, our study identifies the major microbe-responsive enhancers of the *upd3* gene and sheds light on the complexity of the gene regulatory network required in ECs to regulate tissue homeostasis and stem cell activity in the digestive tract.

## Introduction

The digestive tract is uniquely challenged by its high degree of exposure to the external environment. The transit of nutrients through the gastrointestinal (GI) tract is accompanied by frequent introduction of biotic and abiotic stresses. In particular, digestive tissue is constantly exposed to a high density of microbes, including benign microbiota and invasive pathogens [1]. The gut epithelium performs a multifaceted role in maintaining the barrier between the host and its environment through immune responses and the maintenance of a continuous cellular monolayer [2], while digesting and absorbing nutrients. Preservation of epithelial integrity in the GI tract requires continual tissue turnover by coordinated shedding of epithelial cells along with division and differentiation of intestinal stem cells (ISCs) [1,3]. Disorders in epithelial regeneration or intestinal immunity lead to intestinal maladies including inflammatory bowel disease (IBD) and colorectal cancer [4]. Cytokines, which are central to gut homeostasis, are produced by epithelial and immune cells to properly orchestrate immune and repair responses [2,3]. The control of cytokine signaling in the digestive tract is complex, and characterizing the regulators of cytokine expression is a critical step towards fully understanding the mechanisms underlying intestinal homeostasis.

*Drosophila melanogaster* has emerged as a powerful model to study gut homeostasis, epithelial immunity and ISC regulation [1,5], and acts as a model for intestinal infection and pathology [6]. Like the mammalian intestine, the midgut of *Drosophila* contains ISCs that divide and differentiate to replace the absorptive, polyploid enterocytes (ECs) and secretory enteroendocrine cells (EEs) [5]. During division, midgut ISCs self-renew and give rise to a pool of transient, differentiating precursor cells called enteroblasts (EBs), which terminally differentiate into ECs. Similarly, EE cells are replaced via ISCs that divide and give rise to pre-EE progenitors [7]. Also like the mammalian intestine, the *Drosophila* midgut is regionalized. Specifically, it can be divided into five main regions: the cardia (at the foregut-midgut junction), R1 and R2 composing the anterior midgut, R3 also known as the copper cell region, and R4 and R5 that constitute the posterior midgut [8,9].

In response to infection by microbial pathogens or, to a lesser extent, ingestion of dietary microbes, the midgut activates multiple layers of innate immunity. Among these are the induced synthesis of reactive oxygen species (ROS) by the NADPH oxidases Dual oxidase (Duox) and NADPH oxidase (Nox), and the production of antimicrobial peptides under the regulation of the immune deficiency (Imd) and JAK-STAT pathways [10–13]. Imd pathway activation is triggered by the detection of bacteria via peptidoglycan recognition receptors (PGRP-LE and PGRP-LC) [14,15] while JAK-STAT pathway activation results from the expression and secretion from the gut epithelium of *Drosophila* IL-6 family cytokines: Unpaired 3 (Upd3) and Unpaired 2 (Upd2) [16].

In addition to immune activation, enteric infections also stimulate EC delamination and tissue turnover resulting in ISC-dependent tissue repair [12,17,18]. This regenerative process has been shown to depend strongly upon the activation of multiple pathways in progenitor cells, including the Hippo, Wingless, JAK-STAT and EGFR pathways [13,17,19,20]. Bacterial infection, as well as genetically induced apoptosis in ECs, triggers the transcription and secretion of Upd3 in ECs and EBs [13,17], which subsequently initiates a homeostatic feedback loop and ultimately activates ISC-mediated regeneration. The Upd cytokines activate the JAK-STAT pathway in progenitor cells and visceral muscles, which in turn stimulates the release of epidermal growth factors (EGFs) by these cells [19,21,22]. Upd3-dependent secretion of the Epidermal Growth Factors (EGFs) Vein from visceral muscle and Spitz from EBs stimulates the EGFR pathway in ISCs to promote proliferation. Upd3-mediated JAK-STAT activity is also required to promote rapid EB differentiation, thus accelerating epithelium turnover upon infection [13,17]. Cytokines, such as Upd3, therefore act as master regulators of intestinal homeostasis, as they are both required and sufficient to trigger immunity and tissue repair. Accordingly, the loss of Upd3 increases susceptibility to enteric infections, while ectopic induction of Upd3 induces dysplastic lesions in the gut [13,16]. However, a detailed knowledge of upstream enteric stress sensors as well as the downstream transcriptional regulatory network controlling Upd3 production in ECs remains elusive.

In this study, we initiated analysis of the transcriptional regulation of *upd3*, the primary cytokine responsible for inducing ISC proliferation and midgut renewal. We first identified two microbe-responsive enhancer sequences in the *upd3* gene that direct its expression in ECs, and an additional enhancer that regulates *upd3* induction in progenitor cells. A subsequent EC-specific RNAi knockdown screen of all the *Drosophila* transcription factors (TFs) was performed to determine which TFs govern the activity of the central infection-responsive enhancer region. From this screen, we identified 39 TFs required for enhancer induction, and 103 TFs that triggered aberrant induction when knocked down. This study was complemented by an *in vitro*, yeast one-hybrid screen as well as bioinformatic analyses of the enhancer sequence to identify TFs that may act as direct regulators of *upd3* expression. Notably, we identified the Yorkie (Yki)/Scalloped (Sd) complex, the AP-1 complex (D-Jun and D-Fos), Mad and Snail (Sna) as key regulators of *upd3* transcription. We proceeded to explore the upstream regulatory pathways that control the activity of these major TFs. We determined that transcriptional induction of *upd3* in ECs requires the Mitogen Activated Protein Kinases (MAPKs) p38b and D-ERK, downstream of Src oncogene (Src) Family Kinases (SFKs) and Raf, which converge on AP-1 activation. Surprisingly, the Stress Activated Protein Kinase (SAPK) cascade seems to be necessary for only a minimal portion of AP-1 function in ECs. In addition, a Misshapen (Msn)-Warts (Wts)-Yki/Sd pathway, independent of Hippo (Hpo), is essential for full *upd3* expression. Finally, we found that the Decapentaplegic (Dpp) pathway is also required for *upd3* induction in ECs. Altogether, these results improve our understanding of the complex regulation of midgut tissue renewal by identifying the key TFs and pathways that control cytokine signaling in the intestinal epithelium in response to infection.

## Results

### Upd3 transcription is regulated by a combination of microbe-responsive, cell-specific and region-specific enhancers

Upon oral infection by entomopathogenic bacteria like *Erwinia carotovora ssp*. *carotovora 15* (*Ecc15*) or *Pseudomonas entomophila* (*Pe*), Upd3 acts as a signal to trigger antibacterial and reparative host responses [17,23]. We characterized this response through RT-qPCR measurements of midgut *upd3* expression, taken over the course of a week following ingestion of *Ecc15* or *Pe*. We found that *upd3* transcription was strongly induced in response to ingestion of these pathogens and peaked between 8-24h post-infection before returning to basal levels within 96h (S1A and S1B Fig). In addition, peak expression of *upd3*, as well as the time that it takes to return to basal expression, increases with bacterial dose (S1A and S1B Fig). These results demonstrate that *upd3* is regulated by infection at the transcriptional level and varies with the amplitude of the given threat.

**Fig 1:**
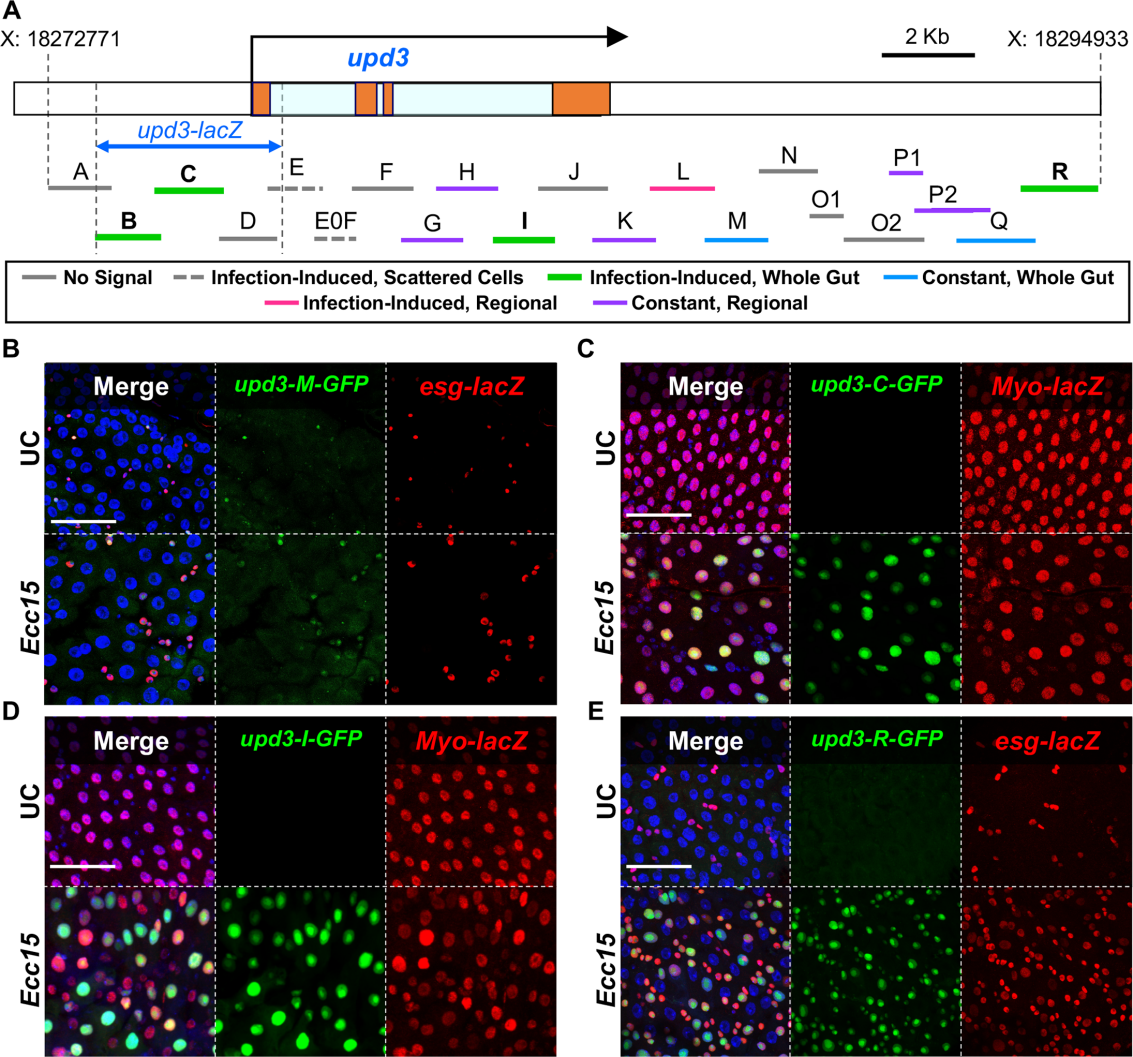
The *upd3* gene is regulated by cell-specific, region-specific and infection-responsive enhancers. (A) Schematic of the *upd3* gene and the 21 overlapping sequences used to create GFP reporter lines. The *upd3* exons are represented by orange blocks and the introns are light blue. Putative enhancer regions have been color coded by their ability to drive GFP expression as follows: Solid Grey–no midgut signal, Dashed Grey–infection induced signal in scattered cells, Green–infection-induced signal throughout the gut, Blue–constant signal throughout the gut, Pink–infection induced signal in a specific midgut region, Purple–constant signal confined to a specific midgut region. (B) Enhancer region M drives an unvarying GFP signal in *esg-lacZ* expressing cells (ISCs and EBs) in all regions. (C, D) Both the C and I enhancer region sequences drive GFP in an infection-inducible manner, specifically in *Myo-*positive cells (ECs) throughout the midgut. (E) Enhancer region R drives infection-induced GFP expression in *esg*-positive cells (ISCs and EBs). (B, C, D, E) Confocal microscopy images taken at 40x magnification with four color channels. DAPI stained nuclei in Blue, GFP in green and antibody stained β-Gal in red. Scale bars are 50μm.

As an initial step to characterize *upd3* regulation in the digestive tract, we sought to identify the key enhancer regions that control its expression, especially its induction in response to pathogens. To this end, we generated twenty-one GFP transcriptional reporters covering the entire *upd3* locus. Overlapping fragments of ~1–1.5Kb were cloned upstream of a GFP reporter, starting from 4.2Kb upstream of the *upd3* start site and ending 7.3Kb downstream of the gene. Reporters were designated *upd3-A-GFP* through *upd3-R-GFP* (Fig 1A, S1 Table). We first evaluated the transcriptional activity of these reporters both in unchallenged (UC) and orally infected flies. Seven lines gave no detectable signal in the digestive system under any condition (enhancers A, D, F, J, N, O1, O2, see Fig 1A). The remaining enhancer regions were divided into five categories based on their expression profile: seven enhancer regions drove GFP expression constitutively, with little change in response to infection by *Ecc15*. *1)* For five of these lines, the signal was limited to specific regions of the gut, including the foregut and hindgut (*upd3-H-GFP*), the foregut only (*upd3-K-GFP*), the hindgut only (*upd3-P1-GFP*), and the copper cell region (*upd3-G-GFP* and *upd3-P2-GFP*) (S1C Fig). *2)* The remaining two constitutive enhancer regions (*upd3-M-GFP* and *upd3-Q-GFP*) are active throughout the midgut in populations of small cells (S1D Fig). Interestingly, cells expressing *upd3-M-GFP* accumulate upon infection (Fig 1B). Overlap of the *upd3-M-GFP* signal and immunostaining of the progenitor marker *esg-lacZ* [24] revealed that the *upd3-M-GFP* reporter is specific to ISCs and EBs, and that the increase in total signal upon infection is thus secondary to progenitor cell proliferation (Fig 1B). *3)* Two additional enhancer regions (*upd3-E-GFP* and *upd3-E0F-GFP*) drove GFP expression in sporadic ECs of the R2 and R4 midgut segments upon infection (S1E Fig). *4)* One enhancer drove inducible *upd3* expression only in the salivary glands (*upd3-L-GFP*, S1F Fig). *5)* Finally, we identified four infection-responsive enhancer regions, which show little or no GFP signal in UC conditions, but are activated upon infection: these include two overlapping regions of the *upd3* promoter (regions B-C), region I, and region R (Fig 1A). Enhancer lines *upd3-B-GFP*, *upd3-C-GFP*, and *upd3-I-GFP* express GFP exclusively in ECs during infection, as shown by co-immuno-staining of GFP and the EC marker *Myo-lacZ* (Fig 1C and 1D and S1G Fig). The *upd3-I-GFP* signal was stronger in the copper cell region and less consistent in the rest of the midgut. In contrast, *upd3-R-GFP* shows activity upon infection only in the ISC and EB cells, marked by *esg-lacZ* (Fig 1E). Of note, the expression patterns identified in our study recapitulate the known *upd3* signaling dynamics in the gut, including induction in ECs and progenitor cells upon stress [13,17,23], as well as robust local expression in the middle midgut and in the cardia [8], suggesting that we adequately captured the complexity of *upd3* regulation. Altogether, these results indicate that *upd3* expression is controlled by several classes of enhancers, including microbe-responsive and region and/or cell type-specific regulators.

### Microbe-responsive *upd3* enhancers are stress-activated enhancers

**Fig 2:**
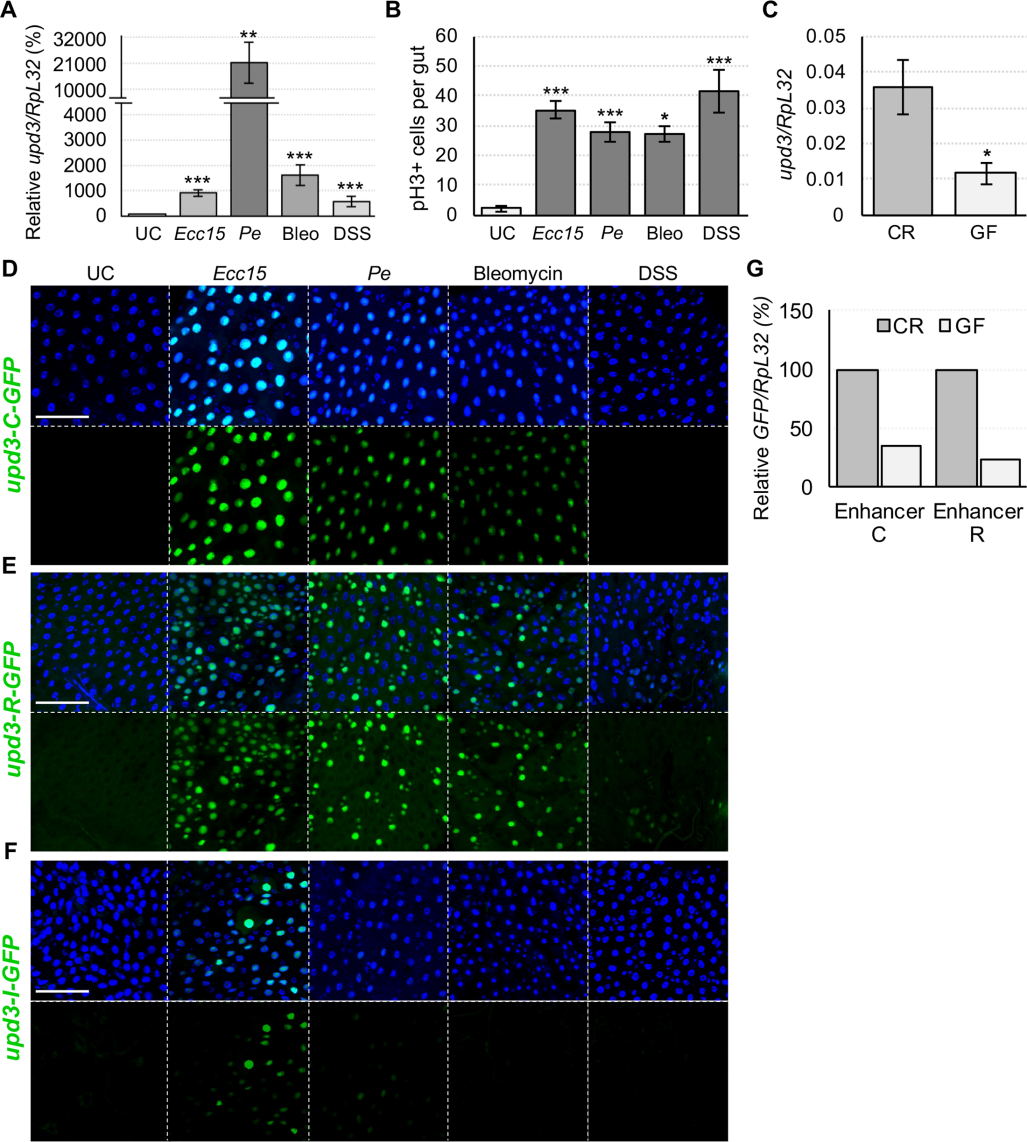
Bacterial infection, stress and the microbiota induce *upd3* through distinct enhancers. (A) RT-qPCR measured *upd3* expression is significantly induced by *Ecc15* and *Pe* infection, as well as bleomycin (bleo) treatment and DSS. (B) ISC proliferation, measured by phospho-Histone H3 (pH3) immunostaining, is triggered in response to ingestion of harmful bacteria (*Ecc15* and *Pe*) and chemical stressors (bleo and DSS). (C) RT-qPCR measurements of *upd3* transcription in the gut of germ-free (GF) flies shows reduced expression compared to their conventionally reared (CR) counterparts. (D, E) Confocal imaging shows that *upd3-C-GFP* and *upd3-R-GFP* strongly induce GFP expression in response to all presented stresses, except for DSS treatment. (F) In contrast, enhancer I responds exclusively to *Ecc15* and marginally to *Pe* infection by GFP induction. (G) Measuring GFP expression in *upd3-C-GFP* and *upd3-R-GFP* flies by RT-qPCR, normalized to the GFP expression in each line under CR conditions, reveals a reduction in basal enhancer C and R activity in GF conditions. Scale bars are 50μm. Statistical significance: mean values of at least 3 repeats are represented ± SEM. *p<0.05, **p<0.01, ***p<0.001 (student’s t-test).

Upd3 acts as a major regulator of intestinal epithelial renewal and its expression is induced by a diversity of enteric stresses, not only limited to bacterial infections [16]. For instance, feeding bleomycin (bleo), which induces gut epithelial cell loss, or dextran sulfate sodium (DSS) that disrupts basal membrane, induces *upd3* transcription in the gut (Fig 2A) and promotes intestinal epithelial turnover (Fig 2B) [25]. Furthermore, basal levels of *upd3* expression and subsequent tissue turnover have been shown to be regulated by the microbiota [16,26]. We confirmed that the guts of germ-free (GF) flies express a lower degree of *upd3* than conventionally raised (CR) flies (Fig 2C). These results suggest that the regulation of *upd3* expression integrates signals from multiple stimuli, including various intestinal injuries and even benign gut microbes.

We next examined whether these diverse stimuli all activate the microbe-responsive enhancers that we had previously identified. To this purpose, we fed *upd3-C-GFP*, *upd3-I-GFP*, and *upd3-R-GFP* flies damaging bacteria (*Ecc15* and *Pe*) and harmful chemicals (DSS and bleo) at doses that trigger comparable epithelium renewal rates. *Upd3-C-GFP* induced GFP expression in response to every treatment except DSS (Fig 2D). Enhancer region R responded to *Ecc15*, *Pe*, bleo, and weakly to DSS by inducing GFP in progenitor cells (Fig 2E). In *upd3-I-GFP* flies, a GFP signal was only detected upon infection with *Ecc15*, and mostly in the copper cell region, while little signal was detected in response to *Pe* and no significant signal was observed in response to bleo or DSS treatment (Fig 2F). Our findings imply that different stresses (i.e. DSS vs other stressors) may be interpreted through distinct cellular mechanisms and thus stimulate cytokine production via separate enhancers. They also suggest that all stressors that affect ECs (*Ecc15*, *Pe*, bleo) stimulate *upd3* expression mainly through enhancer region B-C.

We next investigated whether the infection responsive enhancers C and R also react to the presence of microbiota. To this end, we generated CR and GF *upd3-C-GFP* and *upd3-R-GFP* flies and monitored their levels of GFP (Fig 2G and S2A and S2B Fig). The basal GFP signals of CR flies is already very low with few GFP-positive cells detectable microscopically per midgut, rendering qualitative analysis challenging. We therefore estimated enhancer C and R activity by quantifying GFP levels by RT-qPCR. This revealed a significant reduction in enhancer C and R-driven GFP expression in GF midguts compared to CR ones (Fig 2G). Our results demonstrate that both indigenous and pathogenic bacteria, as well as chemical stressors like bleo, all regulate *upd3* expression through enhancers C and R, albeit to differing degrees. Altogether, these data suggest that enhancers C and R are microbe-responsive and act as stress sensing enhancers.

### *in vivo*, *ex vivo* and *in silico* screens to identify key TFs regulating infection-induced *upd3* transcription

We next aimed to identify the molecular mechanisms that control *upd3* transcription in response to infection. As *upd3* transcription is induced by infection in both ECs (enhancers B-C and I in Fig 1C and 1D, S1G Fig and [12,16,17]) and EBs (enhancer R in Fig 1E and [23]), we began by determining which cell type contributes the most to global *upd3* production in the midgut upon infection. RT-qPCR analysis of *upd3* expression in guts in which *upd3* was knocked-down by RNAi in ECs (*Myo-Gal4*^*TS*^*>UAS-upd3-IR*) or EBs (*Su(H)-Gal4*^*TS*^*>UAS-upd3-IR*) confirmed that ECs are the principal source of *upd3* in the gut upon infection with *Ecc15* or *Pe* (S3A and S3B Fig). In agreement with this, knockdown of *upd3* in ECs strongly reduced ISC proliferative activity (S3C Fig). This suggests that the key enhancers controlling the levels of Upd3 in the gut are those functional in ECs (regions B, C and I). As *upd3-I-GFP* responds only moderately to infection by *Ecc15*, but not *Pe* (Fig 2F), we decided to focus on enhancer regions B and C, which respond strongly to infectious bacteria and cellular stress. To further investigate the importance of the B-C enhancer region in activating *upd3* expression in response to infection we created two new reporter lines, one that comprises the entire *upd3* locus (all enhancers included) and encodes an NLS-GFP-tagged Upd3 protein (full locus) (S4A Fig), and one in which the B-C sequence was deleted from the full locus (full locus–(B+C)) (S4A Fig). While the complete *upd3* sequence was able to direct an infection-induced GFP signal in the midgut, deletion of the B-C region eliminated all signal (S4B Fig), demonstrating that enhancers B and C are central to *upd3* regulation. In addition, quantification of *upd3-C-GFP* signal revealed that the kinetics of GFP induction upon infection is in accordance with total gut *upd3* expression (S1A and S3D Figs). Finally, the promoter of the *upd3* reporter construct, *upd3-lacZ*, which covers regions B and C (Fig 1A), drove a strong and consistent signal in the same cells that are marked by *upd3-C-GFP* (S3E Fig). We conclude that the regulation of enhancer regions B and C (and thus of *upd3-lacZ*) is sufficient to induce *upd3* with a faithful EC expression pattern during enteric infection.

**Fig 3:**
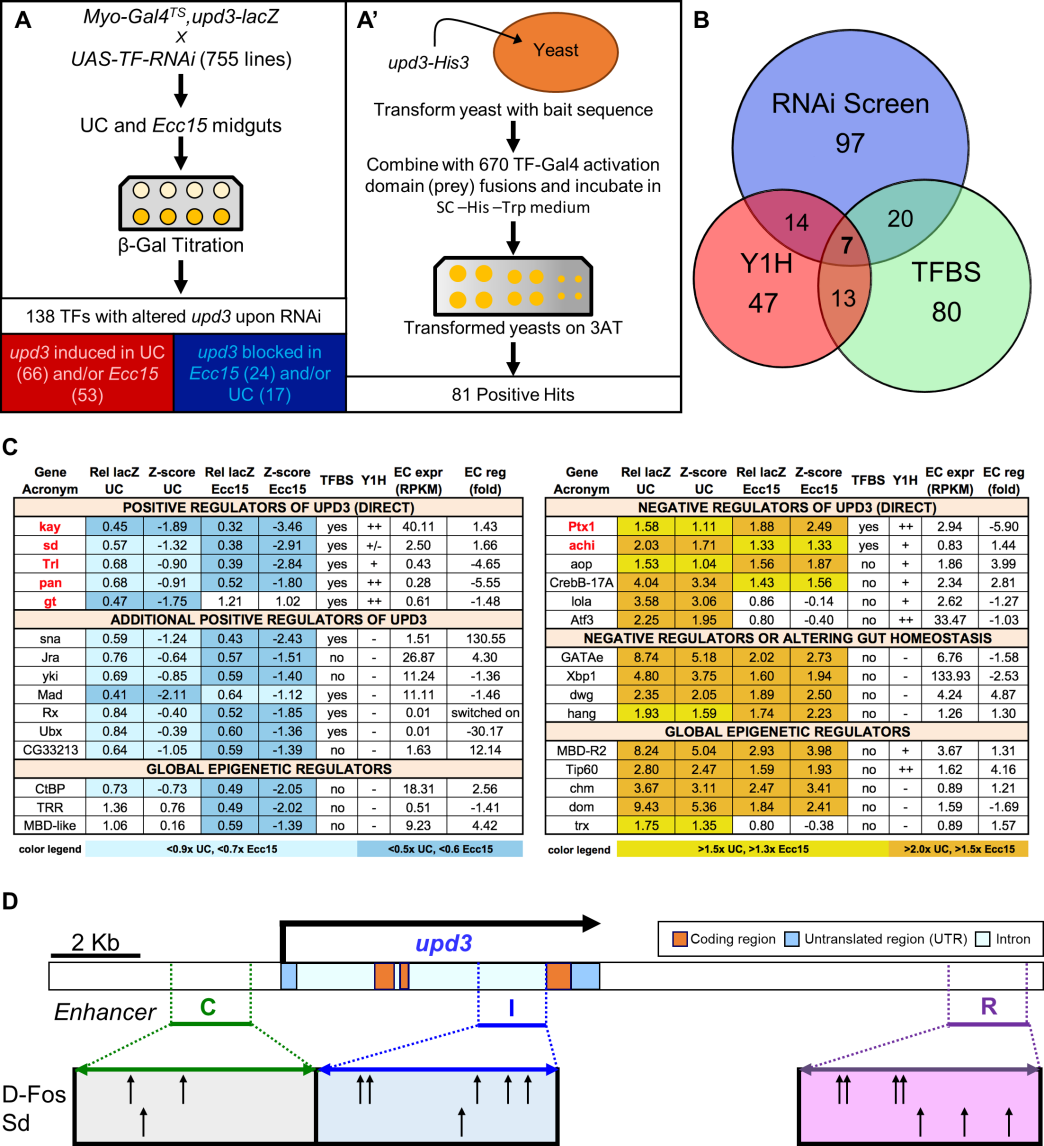
Combination of *in vivo*, *in vitro*, and *in silico* TF screens identifies direct and indirect regulators of *upd3* transcription. (A) Basic schematics of the RNAi (A) and yeast one-hybrid screens (A’) along with the number of positive TF hits for each. (B) Venn diagram displaying the number of positive hit TFs identified by each screen and identified by multiple approaches. (C) Summary table of important TF hits organized by whether they induced or suppressed *upd3* induction, as well as by their TF category: putative direct regulators of *upd3* that likely bind to enhancer regions of the gene, indirect regulators that lack evidence for direct binding potential but have strong phenotypes and probable cause for controlling *upd3*, and epigenetic regulators that may influence *upd3* expression by modifying genomic DNA structure. The seven genes that were positive hits for all three screens are indicated by red text. (D) Schematic representation of D-Fos and Sd binding motifs present in *upd3* enhancer regions C (Green), I (Blue), and R (Purple).

In order to identify the key regulators of *upd3* acting through enhancers B and C, we initiated a comprehensive set of *in vivo*, *ex vivo* and *in silico* screens. First, a functional RNAi screen was performed by driving RNAi-mediated knockdown of 632 TFs (84% of all known and predicted TFs of *D*. *melanogaster*) using all available *UAS-RNAi* transgenic lines of the TRiP collection (Transgenic RNAi Project, Fig 3A) [27]. The Gal4/Gal80^TS^ system (*Myo-Gal4*^*TS*^, *upd3-lacZ*) allowed us to express RNAi specifically in the ECs of adult flies, thus minimizing developmental or systemic side effects. When available, two different *UAS-RNAi* lines were tested (see S2 Table), bringing the total number of lines to 755. Following one week of RNAi induction, five guts were dissected from both unchallenged (UC) and *Ecc15* orally infected flies, and ß-galactosidase enzymatic activity levels were measured as a read-out of *upd3* induction. F1 progeny (*Myo-Gal4*^*TS*^, *upd3-lacZ>UAS-RNAi*) with *upd3-lacZ* activity that was, compared to controls, increased or decreased by 40% upon infection and/or increased or decreased by 50% in UC conditions (see methods section and S5A and S5B Fig) were selected as positive hits. We further estimated the strength of the positive hit phenotypes by calculating their z-score compared to the entire population of crosses tested under the same conditions (UC or *Ecc15* infected) (S2 Table). Based on these criteria, we identified 149 lines with significantly altered *upd3-lacZ* expression in either challenged or unchallenged conditions. Positive hits were retested at least twice and 138 TFs were found to significantly alter *upd3-lacZ* expression when suppressed (Fig 3A and 3B, S2 Table). Specifically, RNAi against 17 TFs in ECs resulted in reduced basal *upd3-lacZ* in UC flies, and knockdown of 66 TFs increased *upd3-lacZ* under the same UC conditions (Fig 3A and S5C and S5D Fig). Furthermore, 24 TFs seemed required for *upd3-lacZ* expression upon infection while RNAi against 53 TFs increased *Ecc15*-induced *upd3-lacZ* activity (Fig 3A and S5C and S5D Fig). These results indicate that the knockdown of many TFs results in *upd3-lacZ* induction rather than inhibition. This is in agreement with the fact that disrupting gut homeostasis by modulating key TFs such as *GATAe*, *Ptx1*, *Activating transcription factor 3* (*Atf3)*, *X box binding protein-1 (Xbp1)*, either in normal or stressed conditions, can indirectly result in higher expression levels of *upd3* [8]. Based on EC-specific transcriptomic data obtained by Fluorescence-Activated Cell Sorting (FACS) of ECs coupled to RNA-seq, we established that 92% of TFs identified as positive hits by our screen are expressed in ECs (RPKM ≥ 0.1) and 63% of the TFs required for *upd3-lacZ* expression are transcriptionally regulated (fold RPKM induction ≥ 1.5 or ≤ -1.5) upon *Pe* infection (S5E Fig) [28,29]. This indicates that most of the TFs identified as *upd3* regulators by our screen are expressed in ECs and regulated upon enteric infection, and serves as an indirect control of our screen quality. Surprisingly, TFs that alter *upd3-lacZ* expression in basal conditions or upon infection are poorly correlated with one another (R^2^ = 0.24, S5F Fig), suggesting that different mechanisms regulate *upd3* expression in basal homeostasis and upon infection. Interestingly, positive hits in our screen were enriched for TFs involved in animal development and tissue growth rather than stress or immune responses, again suggesting that epithelial morphogenesis and dynamics are critical to *upd3* regulation (S5G Fig). Altogether, our functional genetic screen identified multiple TFs that have the capacity to modulate the expression of *upd3-lacZ*, particularly in response to infection.

RNAi knockdown of TFs in ECs can influence *upd3-lacZ* expression in multiple ways: TFs could be acting via direct regulation of the *upd3* promoter region, indirect regulation through secondary genes or even non-cell-autonomously through changes in gut physiology that subsequently alter *upd3* expression. To complement our RNAi screen and identify the direct regulators of *upd3* transcription, we thus undertook two parallel approaches. First, we performed a yeast one-hybrid screen to assess the direct interaction between the *upd3* promoter and all *Drosophila* TFs (Fig 3A’). This additional screen identified 81 yeast one-hybrid-positive TFs (S3 Table). Among these, 21 (more than 25%) showed altered *upd3-lacZ* expression when knocked down, suggesting a role in *upd3* gene regulation (Fig 3B). To further indicate the binding potential of TFs of interest, an *in silico* search for known TF-binding sites (TFBS) was performed in the same genomic region using the JASPAR and RedFly databases (Fig 3B and 3C and S2 Table) [30,31]. We identified seven TFs that are positive for all three approaches, thus specifying them as direct regulators of *upd3*: *D-Fos* or *kayak*, *sd*, *Trithorax-like (Trl)*, *pangolin (pan)*, *giant*, *Ptx1*, *and achintya (achi*) (Fig 3B and 3C). Knockdown of two of these TFs caused abnormal induction of *upd3-lacZ* (*Ptx1* and *achi*). The five others were found to be required for *upd3-lacZ* expression either basally (*giant*) or both during infection and in basal conditions *(D-Fos*, *sd*, *Trl and*, *pan*) (Fig 3C). Of note, Sd and D-Fos have multiple binding sites in infection-responsive enhancers (Fig 3D), and are critical for *upd3* transcription in both UC and infected conditions (Fig 3C). We therefore propose that these TFs act as direct, master regulators of *upd3* expression in the gut.

Next, we examined TFs that strongly alter *upd3-lacZ* expression upon knockdown, but lack evidence for binding potential to the *upd3* promoter region. These important TFs required for *upd3-lacZ* induction include: Sna, a key regulator of epithelial to mesenchymal transition (EMT); Jra (D-Jun), the partner of D-Fos in the AP-1 transcriptional complex; Yki, the transcriptional partner of Sd in the Hippo pathway; Mad, a transcription factor that mediates TGF-β/Dpp signaling; and one thus far uncharacterized TF (CG33213) (Fig 3C and S2 Table). Surprisingly, we found that the homeodomain TFs, Retinal Homeobox (Rx) and Ultrabithorax (Ubx) are also required for *upd3-lacZ* activity, primarily upon infection (Fig 3C), suggesting these TFs could be involved in tissue repair. Among these TFs, Sna, Mad, Rx and Ubx were not found to bind to the *upd3* promoter by the yeast one-hybrid assay, although there are some binding sites in the *upd3* promoter region for these TFs according to the JASPAR database (Fig 3C). This suggests that there is a possibility that they could act directly. Finally, global regulators of transcription such as the transcriptional corepressor CtBP, the H3K4 methyl-transferase Trithorax-Related (Trr) and MBD-like, a member of the NuRD complex also influenced the regulation of *upd3-lacZ* (Fig 3C).

On the opposite side of the spectrum, we also identified TFs that cause increased *upd3-lacZ* expression when knocked-down. For instance, Ptx1, a master regulator of middle midgut identity, has TFBS sites in *upd3*, interacts with *upd3* in the one-hybrid screen and its knockdown strongly induces *upd3-lacZ* in both UC and infected guts (Fig 3C) [28]. This indicates that Ptx1 could act as a direct negative regulator of *upd3* in the middle midgut. The TFs Anterior open (Aop), Cyclic-AMP response element binding protein-17A (CrebB-17A), Longitudinals lacking (Lola), Atf3, and Achi also show potential to bind to the *upd3* promoter region in our one-hybrid screen and trigger *upd3-lacZ* induction when depleted in ECs. RNAi against *GATAe*, *Xbp1*, *deformed wings* (*dwg*), and *hangover* (*hang*) results in elevated levels of *upd3-lacZ* in both *Ecc15* infected and UC conditions, but the absence of TFBS and association in our one-hybrid screen suggests that this is likely an indirect effect due to disruption of intestinal homeostasis. We also found a distinct set of epigenetic factors that strongly increase *upd3-lacZ* activity when knocked-down. Among these, there are known positive regulators of transcription such as MBD-R2 (NSL complex); the Tip60 acetylase; the histone acetyl-transferase Chameau (Chm), Domino (Dom) of the SWI-SNF complex and Trl of the eponymous TRL complex. In summary, our combination of *in vivo*, *in vitro*, and *in silico* screens allowed us to identify putative direct positive and negative regulators of *upd3* induction, as well as key transcriptional regulators of gut homeostasis.

### Indirect positive regulators of *upd3* include the transcriptional repressor Snail, which is induced in ECs upon infection

**Fig 4:**
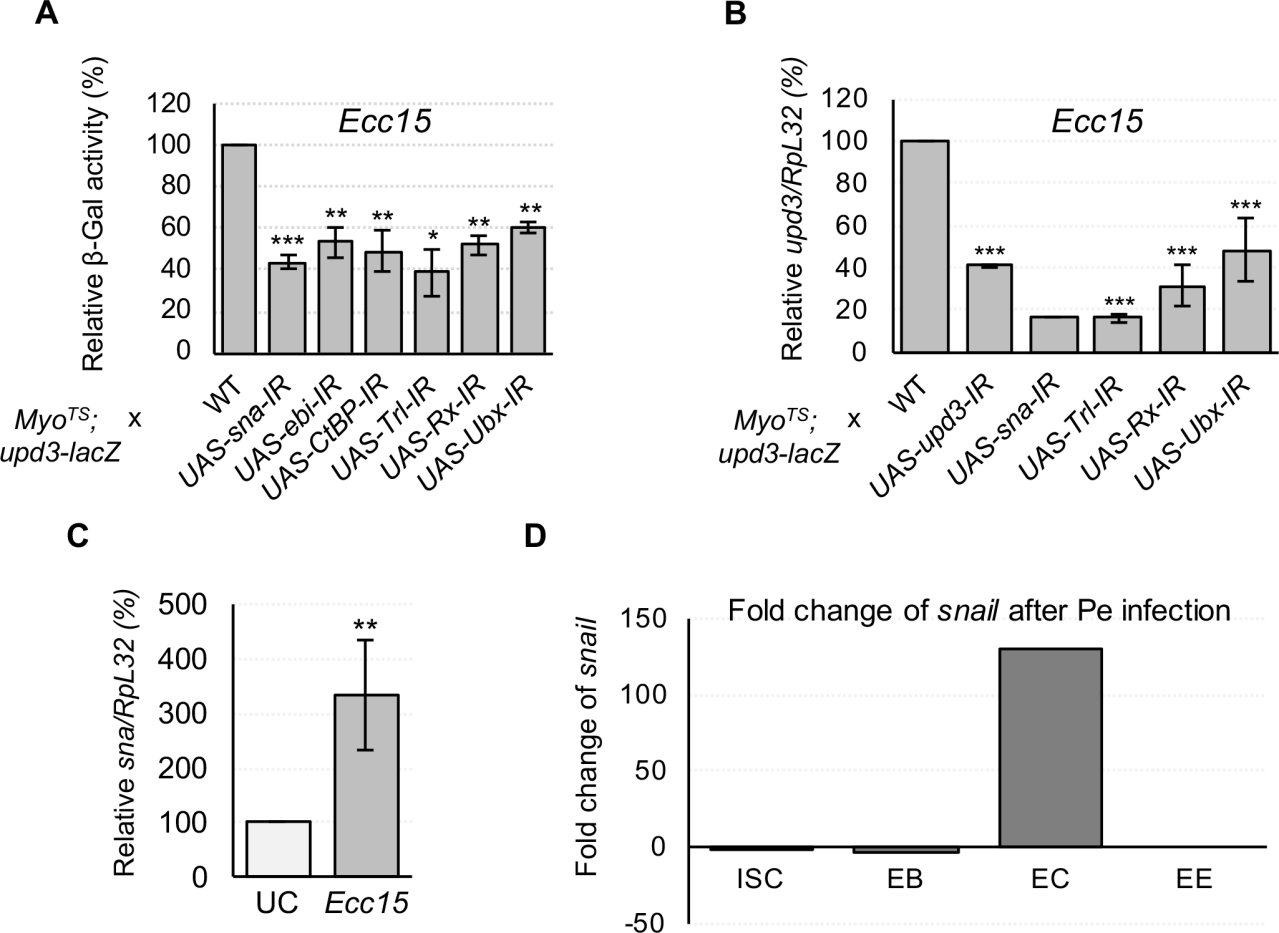
Infection-induced *upd3* expression in ECs requires the indirect functions of Snail and its transcriptional co-repressors, as well as homeodomain TFs and epigenetic regulators. (A) Induction of *upd3-lacZ* by *Ecc15* infection is impeded by RNAi-mediated knockdown of Snail (Sna), its corepressors Ebi and CtBP, the epigenetic regulator Trl, and the homeodomain TFs, Rx and Ubx. (B) RT-qPCR measurements of total midgut *upd3* expression corroborate *upd3-lacZ* results. (C) RT-qPCR measurements of *sna* expression reveal that the gene is transcriptionally upregulated in the midgut following *Ecc15* infection. (D) Cell-specific midgut RNA-Seq data reveals that *sna* is transcriptionally induced specifically in ECs during oral infections by *Pe*. Statistical significance: mean values of at least 3 repeats are represented ± SEM. *p<0.05, **p<0.01, ***p<0.001 (student’s t-test).

Among our positive hit TFs that are strongly required for *upd3-lacZ* induction, we took note of Sna, as well as the homeodomain TFs, Rx and Ubx, and the epigenetic regulator, Trl. Despite the fact that Trl was the only one with a yeast one-hybrid predicted TFBS, knockdown of any of these TFs blocked infection-induced *upd3-lacZ* activity by 40% or more (Fig 3C and Fig 4A). RT-qPCR measurements of *upd3* mRNA levels upon *Ecc15* infection further confirmed the requirement of these TFs for proper *upd3* transcriptional upregulation (Fig 4B).

Sna classically acts as a repressor of transcription [32,33], suggesting that its positive effect on *upd3* expression is indirect. We further confirmed that EC-specific RNAi against CtBP or Ebi, the co-repressors recruited by Sna to mediate transcriptional repression [34,35], also suppressed *upd3-lacZ* activity during *Ecc15* infection (Fig 4A). It is notable that these phenotypes were found in ECs, despite the fact that Sna has been described as a marker and regulator of progenitors in the *Drosophila* midgut [28]. Surprisingly, we found that Sna itself is transcriptionally upregulated in response to both *Ecc15* (Fig 4C) and *Pe* (Fig 4D) infections. In addition, most of its upregulation occurs in ECs (Fig 4D). Altogether, our results suggest that, in response to infection, Sna is upregulated in ECs, and in turn promotes *upd3* upregulation through an indirect mechanism.

### The Hippo pathway controls *upd3* induction in response to infection through the TFs Yorkie and Scalloped

The Hippo pathway consists of a kinase cascade resulting in the phosphorylation of Wts, which in turn phosphorylates and inhibits the transcription factor Yki [36]. When released from phosphorylation-induced restraint, Yki is transported to the nucleus, where it dimerizes with other TFs to promote transcription of target genes [37]. Hippo regulation plays an important role in tissue regeneration and growth. In addition, Yki has been shown to control epithelium turnover, acting cell-autonomously in ISCs via a Hpo/Wts/Yki pathway and non-cell-autonomously in EBs via the Msn/Wts/Yki pathway [38].

**Fig 5:**
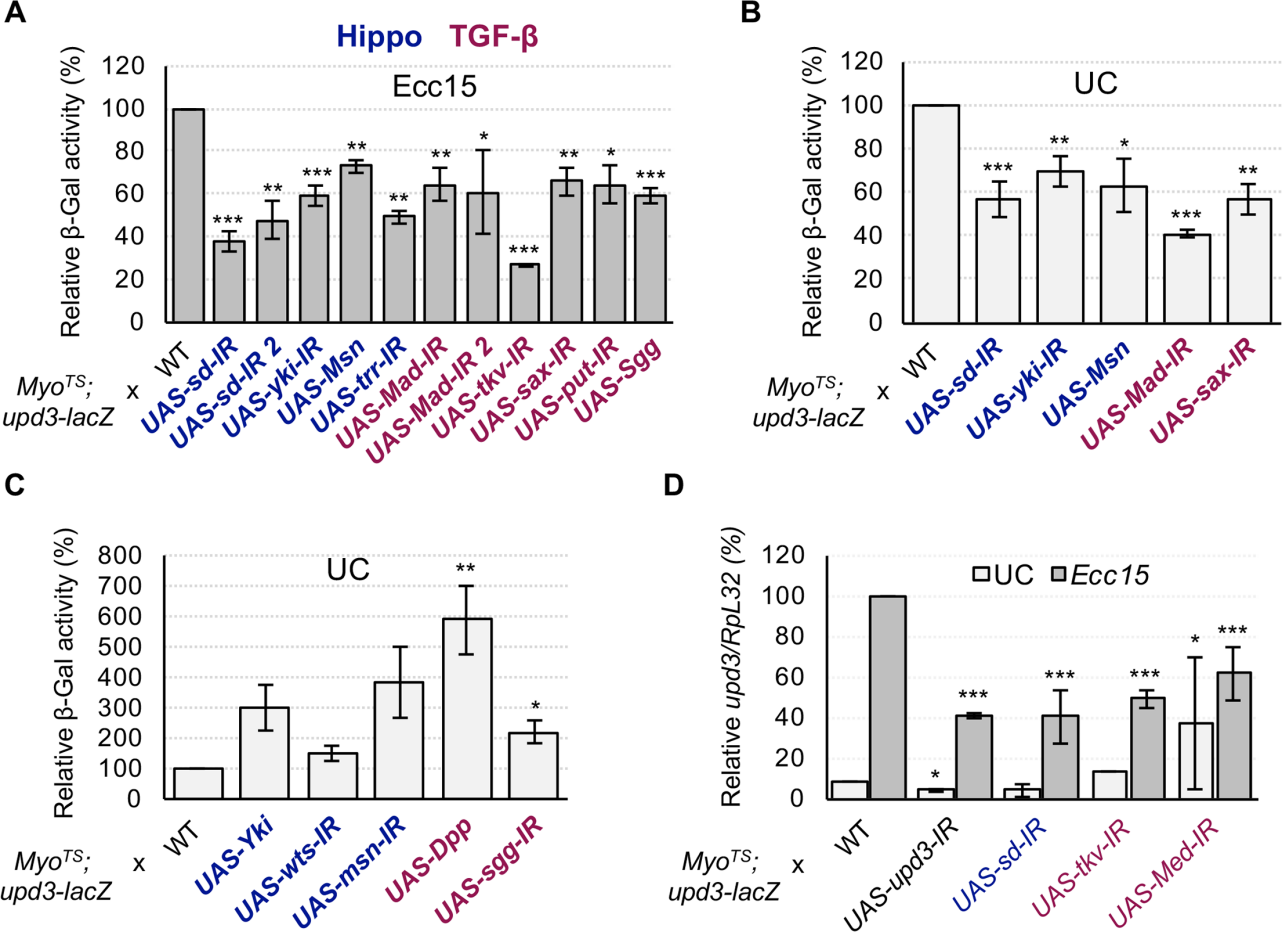
Infection-induced expression of *upd3* in ECs requires the Hippo and Dpp pathways. (A-C) Measurements of midgut *upd3-lacZ* activity under *Ecc15* infected and UC conditions during EC-specific knockdown or overexpression of Hippo and Dpp pathway components. Depletion of the Hippo TFs *sd* or *yki*, or overexpression of an upstream inhibitor (Msn) blocks basal and infection-induced *upd3-lacZ* expression. Likewise, knockdown of *trr*, an epigenetic enhancer of Yki/Sd activity, also inhibits infection-induced *upd3-lacZ*. Alternatively, overexpression of Yki or knockdown of its upstream inhibitors *wts* and *msn* is sufficient to induce *upd3-lacZ*. Knockdown of the Dpp pathway TF *Mad*, either of the three Dpp pathway receptors, *tkv*, *sax*, or *put*, or overexpression of the *Mad* inhibitor, Sgg all blocked *upd3-lacZ* activity. Overexpression of Dpp itself or knockdown of *sgg* induced *upd3-lacZ*. (D) RT-qPCR was used to directly measure *upd3* transcription levels, and confirm that the function of the Hippo and Dpp pathway TFs are required for *upd3* induction. Statistical significance: mean values of at least 3 repeats are represented ± SEM. *p<0.05, **p<0.01, ***p<0.001 (student’s t-test).

As previously mentioned, Yki and its partner Sd were found in our TF RNAi screen to be required in ECs for *upd3* transcription in both basal and *Ecc15*-infected conditions (Figs 3C, 5A and 5B). In addition, Sd was found to interact with the *upd3* promoter by yeast one-hybrid, suggesting that the Hippo pathway may be directly involved in basal and infection-induced *upd3* expression. We also noted that Trr, a major constituent of the TRR histone H3 lysine 4 (H3K4) methyltransferase complex, and Trl, which are both required for full Yki-Sd mediated transcription [39,40], are also required during infection for *upd3-lacZ* induction (Fig 5A). Conversely, overexpressing Yki, or knockdown of either *wts* or its activator, *msn*, in ECs was enough to induce the transcription of *upd3-lacZ* (Fig 5C). However, RNAi mediated depletion of *hpo*, which encodes another Wts phosphorylating kinase, had no significant effect on *upd3-lacZ* (S6 Fig). Finally, overexpressing *msn* in ECs inhibited usual *upd3-lacZ* activity in *Ecc15* infected and unchallenged midguts (Fig 5A and 5B). We confirmed the requirement of the Hippo pathway TF, Sd, for *upd3* transcription in ECs during enteric infection by RT-qPCR (Fig 5D). Our results suggest that the Hippo pathway, which has been shown to be important for *upd3* regulation under basal conditions and in response to abiotic stress [41,42], is additionally required in ECs for *upd3* expression in response to oral infection by *Ecc15*.

### The TGF-β/Dpp pathway is required for *upd3* induction in response to infection

The TGF-β/Dpp pathway has emerged as a major regulator of intestinal homeostasis in *Drosophila*, as it has been found to be involved in diverse processes including ISC proliferation, ISC quiescence, EC differentiation and EC protection [43–48]. Mad, a TF downstream of the Dpp pathway was found in our screen to be necessary for wild-type *upd3-lacZ* levels upon ingestion of *Ecc15* as well as in basal conditions (Fig 5A and 5B). Thus, we explored whether ECs require a fully functional Dpp pathway to regulate the transcription of *upd3*. EC-specific RNAi against the Dpp type-1 receptors, *thickveins* (*tkv*) and *saxophone* (*sax*), or the type-2 co-receptor *punt* (*put*), all decreased infection-responsive *upd3-lacZ* activity (Fig 5A). Furthermore, overexpression of Dpp triggered aberrant induction of *upd3-lacZ* (Fig 5C). We additionally tested the Dpp pathway via manipulation of the glycogen-synthase-3-kinase Shaggy (Sgg), which has been shown to negatively regulate Mad through phosphorylation of linker serines [49]. Overexpression of *sgg* in ECs blocked *upd3-lacZ* induction, while *sgg* knockdown increased *upd3-lacZ* basal activity (Fig 5A and 5C). A role for the Dpp pathway in regulating *upd3* was further supported by RT-qPCR of *upd3* in flies expressing EC-specific RNAi against *tkv* or *Medea* (*Med*), a TF that acts together with Mad [50], as both led to decreased induction of *upd3* upon *Ecc15* infection (Fig 5D). Altogether, our data demonstrate that the Dpp pathway is required for proper *upd3* transcription in response to infection.

### Src-Raf-Dsor1-ERK and Licorne-p38 pathways converge to regulate *upd3* transcription upon infection

**Fig 6:**
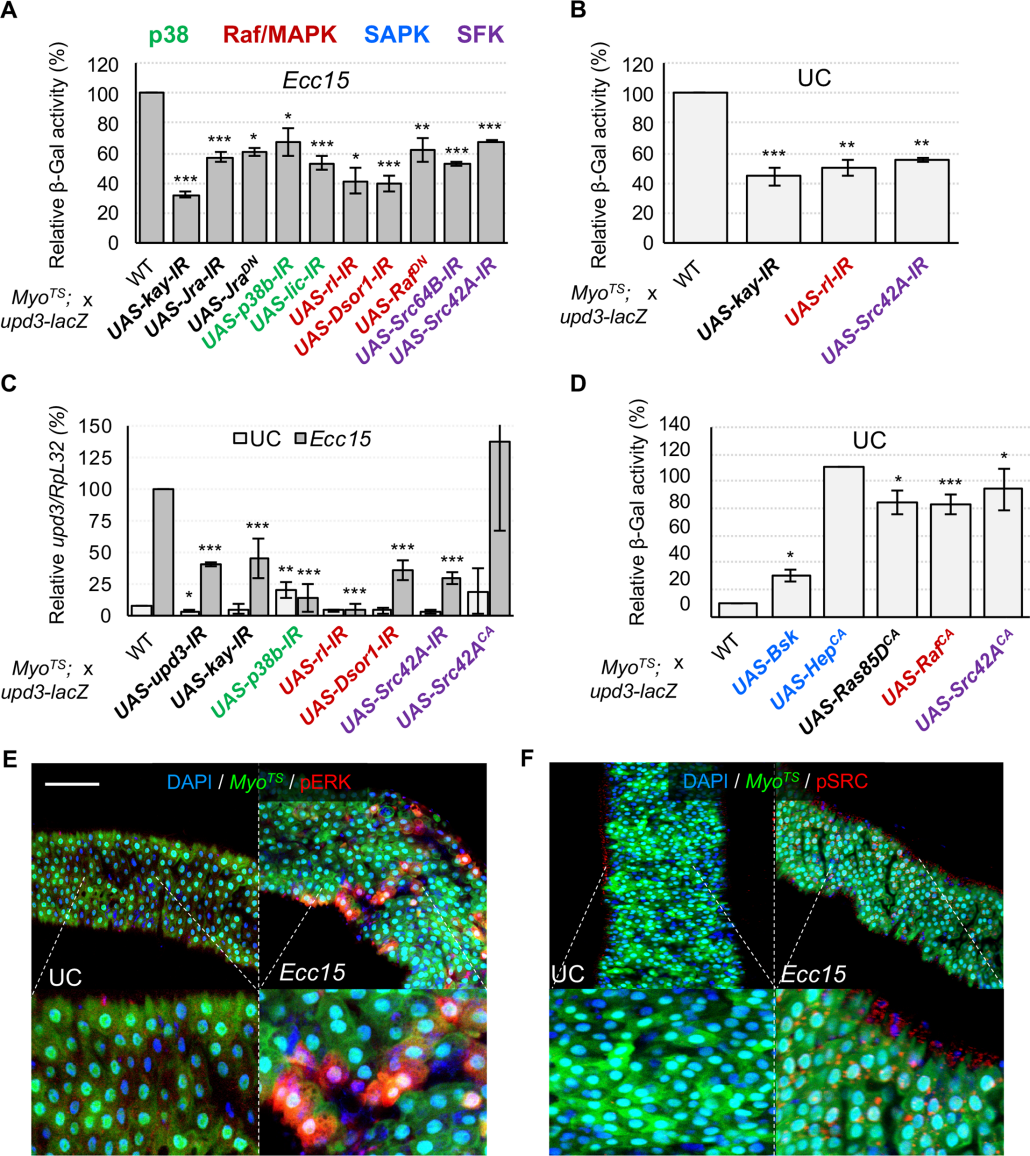
Infection-induced *upd3* expression in ECs requires the TFs D-Jun and D-Fos, activated by upstream Src-MAPK pathways. (A-B) Knockdown by RNAi of multiple constituents of MAPK pathways, as well as Src kinases or the TFs D-Jun (Jra) and D-Fos (Kay) inhibits *upd3-lacZ* activity under *Ecc15* infection or UC conditions. (C) RT-qPCR measurements of total midgut *upd3* expression corroborate *upd3-lacZ* results. (D) In addition to their requirement for *upd3-lacZ* activity, activation of the MAPKs and SFKs can also induce *upd3-lacZ* expression in UC conditions. SAPKs can also induce this activity when stimulated. (E, F) Immunostaining against phosphorylated forms of ERK and Src reveals that these kinases are activated in response to infection in ECs. Scale bar is 100μm. Statistical significance: mean values of at least 3 repeats are represented ± SEM. *p<0.05, **p<0.01, ***p<0.001 (student’s t-test).

D-Fos and D-Jun were among the TFs in our screen that most strongly impacted *upd3-lacZ* activity upon infection. When activated by upstream kinases these two TFs act together as the AP-1 transcription factor complex [51]. D-Fos also interacts *ex vivo* (in our Y1H screen) with the *upd3* promoter, suggesting that AP-1 acts as a direct regulator of *upd3* transcription. Accordingly, RNAi against *D-Fos* or *D-Jun*, or the expression of a dominant negative D-Jun (*UAS-Jra*^*DN*^) significantly decreased *upd3-lacZ* activity (Fig 6A and 6B). As an additional confirmation of these results, we found that RNAi mediated knockdown of *D-Fos* in ECs prevented infection-responsive *upd3* expression as measured by RT-qPCR (Fig 6C).

We next aimed to identify the upstream pathway(s) that regulate(s) D-Fos and D-Jun in response to *Ecc15* infection. Phosphorylation and subsequent activation of the AP-1 complex is carried out by both Stress Activated Protein Kinases (SAPKs) and Mitogen Activated Protein Kinases (MAPKs) [52]. SAPKs and MAPKs act in phosphorylation cascades that result in the activation of terminal kinases such as JNK, Basket (Bsk), p38 and ERK (S8F Fig). It has been previously shown that artificial activation of the *Drosophila* SAPK, Bsk, by overexpression of Hemipterous (Hep) induces *upd3* transcription in the gut, possibly through the activation of apoptosis or by directly regulating AP-1 [17,19]. We first evaluated whether apoptosis is required for *upd3* expression in response to microbes. To this end, we manipulated the expression of caspase and autophagy genes in ECs and measured the resulting *upd3-lacZ* activity. Our results confirmed that promotion of autophagy or apoptosis, by overexpression of *Autophagy-related 1 (Atg1)* or Death regulator Nedd2-like caspase (*Dronc*), respectively, induced *upd3* (S7A Fig). However, inhibiting either pathway by RNAi against *Dronc*, *Death-associated APAF1-related killer (Dark)*, *Atg1*, *Atg7* or *Atg18*, or by overexpression of the caspase inhibitor P35 (*UAS-P35*), had no significant negative effect on *upd3-lacZ* levels during infection (S7B Fig). Furthermore, detection of caspase activity in ECs by the *UAS-Apoliner* system (S7C Fig) [53], in conjunction with immunostaining for *upd3-lacZ*-derived β-galactosidase, revealed that cytokine production during enteric infection is not restricted to ECs with increased caspase activity (S7D Fig). Altogether these data suggest that apoptosis and autophagy are not the key inducers of *upd3* expression upon infection.

We next sought to evaluate the contribution of JNK to *upd3* induction upon infection with *Ecc15*. We first verified whether *Ecc15* infection triggers JNK activation in ECs, via co-immunostaining of the phosphorylated form of JNK and an EC marker (*Myo-Gal4*^*TS*^*>UAS-GFP*) (S8A Fig). In agreement with previous publications, ectopic activation of the JNK pathway in ECs, by overexpressing Bsk or a constitutively active form of Hep, strongly promoted *upd3-lacZ* transcription (Fig 6D). However, EC-specific expression of a dominant negative form of Bsk (*UAS-Bsk*^*DN*^), or knockdown of *bsk* expression, decreased *upd3-lacZ* activity following oral infection by only 20% (S8C Fig). Additionally, RNAi knockdown of *hep* did not decrease *upd3-lacZ* induction significantly (S8C Fig). This suggests that JNK only plays a minor role in *upd3* regulation, and thus additional stress pathways may be responsible for stimulating AP-1 in response to oral bacterial infection.

Another possible candidate for AP-1 regulation is the p38 family of stress responsive MAPKs. The p38 kinases can regulate the AP-1 complex (S8F Fig), and have been shown to be involved in the response to oral infection in *Drosophila* [54]. Immunostaining for phosphorylated p38 kinases revealed a substantial increase in p38 phosphorylation in ECs upon infection (S8B Fig). To investigate the role of the p38 pathway further, we knocked down the three p38 kinases of *Drosophila* (*p38a*, *p38b* and *p38c)*, independently. Only knockdown of *p38b* gave a mild, but significant (p<0.05) decrease in *upd3-lacZ* induction upon infection (Fig 6A). We similarly tested the involvement of the upstream p38 MAPKK, Licorne (Lic), and found that knockdown of *lic* in ECs also blocks increased *upd3-lacZ* transcription in response to oral infection. These experiments suggest that the stress in ECs caused by enteric infection triggers activation of a Lic/p38b pathway that mediates part of the induction of *upd3-lacZ*.

In addition to JNK and p38 kinases, the D-ERK kinase is also able to activate the AP-1 complex (S8F Fig) [51]. Thus, we decided to investigate whether the MAPK/D-ERK pathway could also act upstream of AP-1 to regulate *upd3* upon infection. Immunostaining for the phosphorylated form of Rolled (Rl), the *Drosophila* homologue of ERK, revealed that infection with *Ecc15* triggers D-ERK activation in ECs within two hours (Fig 6E). Furthermore, RNAi knockdown of *rl* in ECs resulted in a strong decrease in *upd3-lacZ* activity upon infection (Fig 6A), suggesting that the MAPK/ERK pathway is necessary for infection-regulated *upd3* induction. MAPKs are activated in a phosphorylation cascade downstream of MAPKKs and MAPKKKs (S8F Fig). Two of the four *Drosophila* MAPKKs (Lic and Hep) were previously tested for a role in *upd3* regulation, and thus we proceeded to test the remaining two: Downstream of raf1 (Dsor1) and MAP kinase kinase 4 (Mkk4). As for ERK, Dsor1 was critical for full induction of *upd3-lacZ* upon infection (Fig 6A). Accordingly, expressing a dominant negative form of the upstream MAPKKK, Raf, in ECs also decreased *upd3-lacZ* regulation by infection, while blocking other MAPKKKs, TGF-β activated kinase 1 (TAK1), Apoptotic signal-regulating kinase 1 (ASK1) and MEKK1, did not (Fig 6A and S8D Fig). Furthermore, constitutively active Raf expression is sufficient to induce *upd3-lacZ* activity (Fig 6D). These data together suggest the possibility of a Raf/Dsor1/ERK pathway that regulates *upd3* expression via AP-1 in response to midgut infection or damage. Activation of Raf by phosphorylation is typically accomplished via Ras, downstream of growth factor receptors (S8F Fig). However, although overexpression of constitutively active Ras is sufficient to induce *upd3* (Fig 6D), blocking Ras itself (S8C Fig) or signaling through the key Receptor Tyrosine Kinases (RTKs) EGFR and PDGF- and VEGF-receptor related (Pvr) (*UAS-Ras*^*DN*^, *UAS-EGFR*^*DN*^, *UAS-Pvr-*^*DN*^) did not impair *upd3-lacZ* activity (S8E Fig). Likewise, RNAi knockdown of the Pvr ligand, PDGF- and VEGF-related factor 2 (Pvf2), had no effect on *upd3-lacZ* regulation. Raf signaling can occur downstream of additional tyrosine kinases, including the Src family kinases (SFKs, S8F Fig) [55,56]. Immunostaining for the phosphorylated form of Src kinases revealed that infection with *Ecc15* triggers Src activation in ECs (Fig 6F). To determine if the Src complex is also required for *upd3* regulation, we knocked down *Src42A* and *Src64B* by RNAi in ECs (Fig 6A). Depletion of either *Src42A* or *Src64B* decreased *upd3-lacZ* induction upon infection. Conversely, the expression of a constitutively active form of Src42A in ECs triggered *upd3-lacZ* induction in absence of infection, suggesting that a Src/Raf/Dsor1/MAPK pathway is sufficient to activate *upd3* transcription. We further confirmed our results by RT-qPCR of *upd3* in response to infection while blocking expression of *Dsor1*, *p38b* and *Src42A* in ECs by RNAi, as well as by activating the pathway by expression of a constitutively active form of Src42A (Fig 6C). In summary, our results demonstrate that multiple kinase cascades (Licorne-p38b and Src/Raf/Dsor1/ERK) are activated in ECs following oral *Ecc15* infection and converge on the regulation of *upd3*.

### Impairment of *upd3* regulatory TFs or their upstream activators in ECs reduces ISC proliferation and compromises adult lifespan

**Fig 7:**
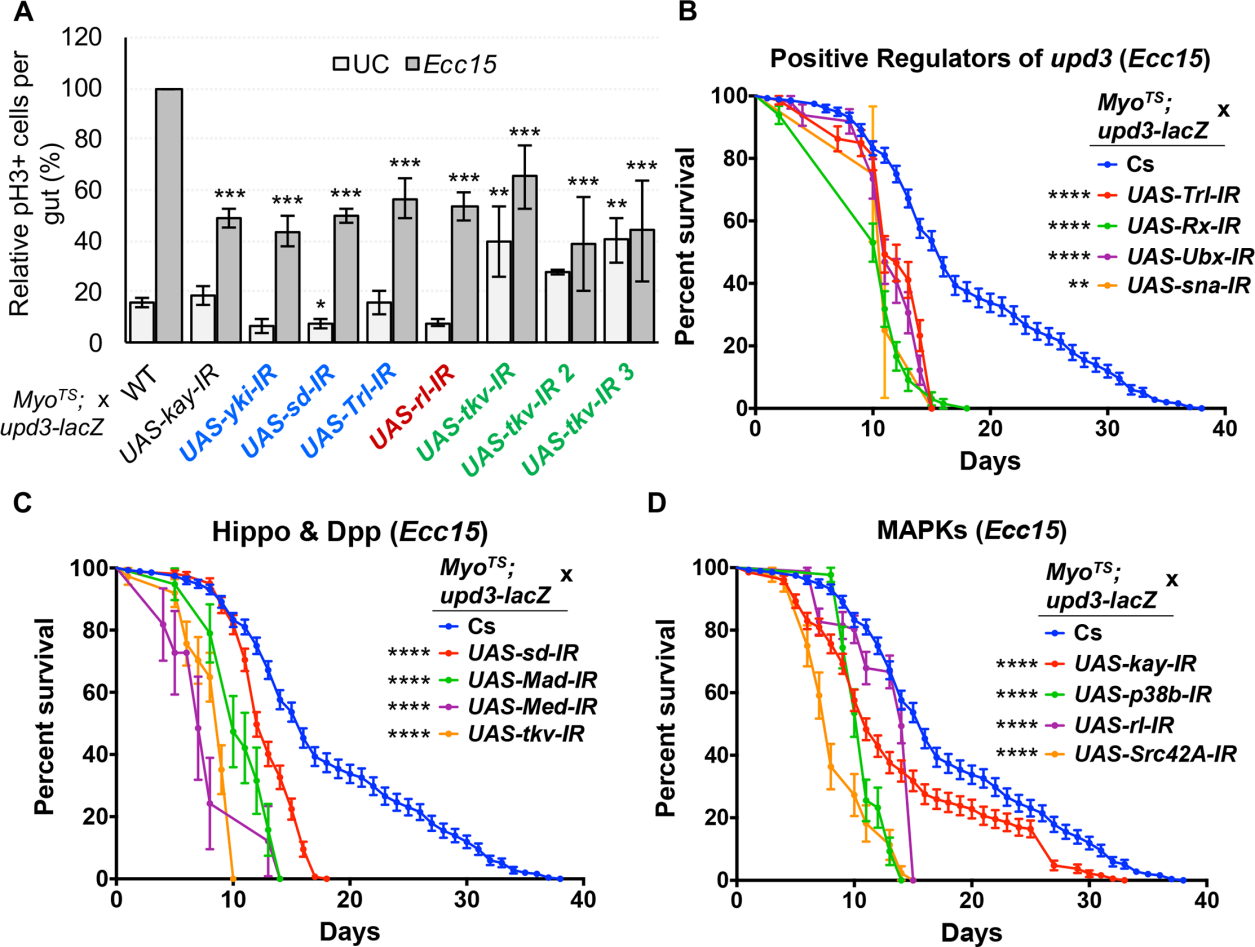
ISC proliferation and survival following *Ecc15* infection are compromised by inhibition of the TFs and pathways that are required for *upd3* induction. (A) Total pH3+ cell counts in unchallenged and *Ecc15* infected guts demonstrate that knockdown in ECs of *D-Fos*, *yki*, *sd*, *Trl*, and *sna* as well as upstream components of the MAPK and Dpp pathways is accompanied by a decrease in ISC mitotic activity. Statistical significance: mean values of at least 3 repeats are represented ± SEM. *p<0.05, **p<0.01, ***p<0.001 (student’s t test). (B-D) Survival curves of flies orally infected with *Ecc15* alongside RNAi-induced knockdown of indirect *upd3* regulators (B), Hippo and Dpp pathways components (C), or MAPK pathway factors (D). Curves represent averaged survival ± SE. Statistical significance: *p<0.0332, **p<0.0021, ***p<0.0002, ****p<0.0001 (Log-rank test).

We next aimed to evaluate the physiological consequences of modulating in ECs the pathways that control *upd3* transcription. The number of mitotically active ISCs (phospho-Histone H3 positive cells) following *Ecc15* ingestion was significantly reduced by knockdown of AP-1 and Sd, as well as the MAPK, Rl, the Dpp receptor, Tkv, and the epigenetic regulator, Trl, using the temperature sensitive, EC specific driver line (*Myo*^*TS*^) (Fig 7A). This suggested that pathways required for EC-derived Upd3 production are required for proper ISC activity upon infection. We therefore monitored the survival of flies expressing EC-specific RNAi against pathway components of the Hippo, Dpp and SFK/MAPK/AP-1 pathways as well as putatively indirect regulators (Sna, Trl, Rx, Ubx) of *upd3* upon infection. These flies had significantly shorter lifespans following *Ecc15* infection compared to wild-type controls, and LT50 values lower than controls by at least two days (Fig 7B–7D, S4 Table). In addition, we also found that, under UC conditions, these knockdown flies have significantly shorter lives than wild-type ones, and correspondingly lower LT50 values (S9A–S9C Fig, S4 Table), implying that the knockdown of these genes, or the subsequent reduction in basal Upd3 levels compromises midgut epithelial homeostasis. We further confirmed our results by altering the expression of our candidate genes in ECs using multiple independent transgenic *UAS-RNAi* lines for each gene and monitoring their survival in both infected and unchallenged conditions (S4 Table). Altogether, our experiments demonstrated that the Hippo, Dpp and SFK/MAPK/AP-1 pathways are required in ECs for survival to oral infection and for normal aging.

## Discussion

The *Drosophila* Unpaired ligands, as well as mammalian type I family cytokines, such as IL-6, play an essential role in activating JAK-STAT and other signaling pathways upstream of tissue renewal. Our study provides insight into the complex regulation of Upd3, a cytokine that is transcriptionally induced in response to pathogenic and endogenous microbes, and initiates immune activation and stem cell proliferation.

### Microbe-responsive enhancers as DAMP sensors

We found that the *upd3* gene is regulated by three classes of enhancers: region-specific, cell-specific and stress/microbe-responsive. This complexity likely reflects the multiple roles of the JAK-STAT pathway in the *Drosophila* midgut, where it acts to stimulate ISC proliferation, promote differentiation and serves as a regional determinant of cell identity, notably in the middle midgut [8,12,17,57]. We propose that the different functions of *upd3* are therefore regulated independently by the diverse enhancer regions we identified. We further identified microbial responsive enhancers that are active either in ECs (B-C and I) or in progenitor cells (enhancer R), supporting a distinct regulation of *upd3* in different cell types. Interestingly, the progenitor-specific enhancer R is the only one to be induced by DSS feeding (and only to a low degree), while the EC specific enhancers B-C and I do not promote transcription in these conditions. It has been speculated that DSS elicits stem cell proliferation through alteration of the basal lamina rather than by direct damage to ECs [25], such as that caused by *Ecc15* infection or by bleo treatment. This suggests that different cell-type specific enhancers allow for induction of *upd3* expression in response to a broad variety of stresses.

The regulation of host gene expression by bacteria in *Drosophila* relies mostly on dedicated pathways, Toll and Imd, that trigger effector induction in response to the detection of microbial patterns (MAMPs), such as bacterial derived peptidoglycan [58]. The microbe-responsive enhancers of *upd3* are activated by both pathogenic and benign microbes, such as *Ecc15* and the gut microbiota, but are also stimulated by toxic chemicals such as bleo or DSS. This result suggests that cytokine production in the gut is primarily triggered in response to damage associated molecular patterns (DAMPs) rather than the detection of microbes alone. Considering that dietary microbes and the microbiota are constantly associated with the gut tissue, triggering perpetual, low-level Imd activation, responding to DAMPs could be a strategy to couple immune activation and tissue repair to the presence of pathogens rather than beneficial or commensal microbes. Accordingly, we found that *upd3* activation is less pronounced by the microbiota than by pathogens. These pathways have been shown to be activated by various stresses and are central to *upd3* regulation in ECs. A major source of stress in response to microbes, is the production of ROS, partly induced by NADPH oxidases Nox and Duox of the host immune response [59,60]. Notably, SAPKs and Src kinases are both sensitive to ROS and their activity is modulated by oxidative stress, indicating that a NADPH oxidase, ROS, Src, SAPK/MAPK axis could be involved in *upd3* regulation. Future work should determine the link between infection-induced ROS, Src/SAPK activation and the control of gut homeostasis.

### *upd3* integrates signals from multiple signaling pathways

We further focused on identifying the key TFs that regulate *upd3* in the midgut. We found that altering the expression of 138 over the 708 *Drosophila* TFs significantly altered *upd3* expression in the midgut. This number is surprisingly high, as it implies that a quarter of *Drosophila* TFs directly or indirectly regulate *upd3* transcription. We interpret this high number as an indication that *upd3* acts as a stress marker, and that any physiological alteration in the gut will result in a rupture of gut homeostasis and consequently in the induction of *upd3* [8]. We therefore propose that *upd3* acts as a global sensor of gut stress and in turn initiates a stereotypical immune and homeostatic program.

This poses the question of how multiple stresses can converge on the activation of *upd3* transcription. Our results suggest that in ECs, stresses are mostly integrated by one *upd3* enhancer (B-C) that responds to both chemical and biotic stresses. Integration could occur either because all stresses result in one simple damage signal, for instance cell loss in the epithelium, or as a consequence of multiple types of gut damage. Interestingly, the TFs altering *upd3* expression in basal and infected conditions are not the same, indicating that different cascades regulate *upd3* expression under different conditions. Upon infection, our data show that the Dpp, Hippo, SAPK and MAPK pathways are all involved in the regulation of *upd3*. We therefore propose a model in which the diverse transcriptional regulation of *upd3* is required for its multiple roles in homeostatic regulation.

The different transduction pathways we identified all respond to different cues. We find that the Dpp pathway is likely involved in the activation of enhancer B-C in the *Drosophila* midgut. The Dpp pathway is furthermore essential for EC differentiation, growth, survival to infection, and injury-induced Dpp negatively controls midgut homeostasis [43,45]. Upon enteric infection, the Dpp pathway displays complex behavior. In an early response, Dpp released from hemocytes has been shown to stimulate ISC proliferation, but in a second phase, the Dpp pathway promotes the reestablishment of a quiescent state in these same cells [61]. Our results suggest that upon infection with *Ecc15*, the Dpp pathway also plays a role in ECs by promoting *upd3* transcription, which could synergize with the early proliferative role of this pathway in ISCs. It remains unclear whether Dpp acts directly or indirectly on the *upd3* promoter. We identified Mad and Med as required for *upd3* expression, and TFBS for Mad are found in the promoter region of *upd3*; however, our yeast one-hybrid screen did not detect a direct interaction between these two components.

We did find evidence of direct regulation of the *upd3* gene by transcription factors downstream of the Hippo pathway and SAPK/SFK/MAPK cascades. The Hippo pathway regulates ISC proliferation in the midgut both cell-autonomously and non-cell-autonomously [42,62,63]. The upstream regulators of Hippo signaling remain uncharacterized in the midgut, but the MAPKKKK Msn has been shown to control Wts in progenitor cells [64]. Our data suggest that the Yki/Sd complex directly regulates *upd3* in ECs upon infection, and that Msn, but not Hpo, is involved in that process. We furthermore identified D-Fos and D-Jun (AP-1 complex) as direct regulators of *upd3* transcription, acting downstream of Src-Raf-Dsor1-ERK and Licorne-p38b kinase cascades. Stress responsive kinases, as well as SFKs, are key regulators of AP-1 [55]. It remains unclear whether the upstream stimuli inducing SAPK/SFK/MAPKs to regulate *upd3* upon infection include oxidative stress, cytoskeletal modification or a combination of both, but all these stimuli occur upon infection and are possible candidates. We propose that the role of SFKs, MAPKs and SAPKs in the regulation of cytokine expression and cell proliferation is conserved across organisms. Indeed, AP-1 and these conserved pathways have been demonstrated to have an important role in the regulation of cytokine secretion and tumorigenesis [65,66]. Src kinases have been previously shown to be important for wound healing in multiple models, potentially downstream of ROS production [67,68], suggesting that conserved pathways are used in both tissue repair and gut regeneration. Interestingly, the pathways we identified in our study are known to work cooperatively in other systems. For example, mammalian JNK kinases are capable of phosphorylating YAP (Yki homologue), and can inhibit multiple constituents of the Hippo pathway during tumorigenesis [69]. In addition, mammalian Src has been shown to regulate YAP during inflammation [70]. Finally, it was recently found that binding sites for Yap/Taz/Tead (Yki/Sd in *Drosophila*) and AP-1 are associated genome-wide with enhancers of genes involved in oncogenic growth. Altogether, these results and our own suggest that the SAPK/SFK/MAPK pathways in coordination with Hippo and TGF-β pathways work together in a conserved regulatory network that controls tissue growth and repair.

### Cell loss, Upd3 regulation and tissue renewal

The maintenance of gut tissue homeostasis relies on the induction of ISC proliferation to compensate for the loss of cells in the epithelium in a homeostatic feedback loop. A simple model of homeostasis would hold that cell death directly triggers *upd3* expression and subsequent ISC proliferation in a coupled manner. In agreement with this model, induction of apoptosis in ECs is sufficient to induce *upd3* expression and trigger ISC proliferation [17]. However, a recent study using oral infection with a low dose of pathogenic bacteria in *Drosophila* demonstrated that cytokine-induced ISC proliferation can be elicited even by infections that do not induce epithelial cell death [71]. This indicates that the coupling of ISC proliferation with cell loss is not complete. In agreement with these results, we found that neither apoptosis nor autophagy alone appear to be necessary for *Ecc15*-induced *upd3* expression. Rather, the results of Loudhaief *et al*. (2017) and our study suggest that cytokine signaling results from stress detection rather than cell death, and that regenerative processes can occur independently of apoptosis [71]. This is also in agreement with the fact that the gut microbiota, which induces basal levels of epithelial stress but does not induce massive cell death in the gut, also stimulates basal cytokine production [3,13,16,26]. Pathways such as Hippo regulate both cell death and apoptosis, as well as cytokine production in the gut. We therefore propose that coupling between cell death and cell renewal is a consequence of cross-talk between regulatory pathways, rather than renewal as a direct consequence of cell death.

Another hypothesis is that cell loss without death is coupled to tissue repair. Accordingly, infection induces the loss of ECs from the epithelium prior to anoikis [19]. It is therefore possible that EC delamination, rather than death, is a key signal for regeneration as evidenced by the observation that loss of EC contact with the basal lamina of the midgut epithelium can trigger Upd3 production [72]. EMT is a process of tissue morphogenesis reminiscent of cell delamination, in which epithelial cells detach and are extruded from the epithelial sheet whereupon they migrate as loosely associated mesenchymal tissue. Curiously, our study shows that the transcription factor Sna, a main regulator of *Drosophila* EMT and a marker of progenitor cells in the midgut, is both transcriptionally induced in ECs upon infection and required for *upd3* transcription [8,73]. Sna’s role as a negative regulator of transcription implies that this phenotype is likely a secondary effect. We thus propose that *upd3* expression may be downstream of Sna-dependent, EMT-like shedding of ECs in response to enteric stresses. In such a scenario, cell loss would require an EMT like regulation in ECs and indirectly trigger *upd3* transcription. Epithelial structure and tension modulated by delamination could also result in Src and Hippo pathway activation, and ultimately in *upd3* induction. Future work will determine how ECs are extruded from the epithelial sheet and how cell loss modulates Upd3 production.

### Regulatory networks are reused in multiple epithelial cell types to coordinate tissue repair

The regulatory pathways that we find upstream of *upd3* transcription in ECs appear to be the same pathways required in ISCs to control their proliferation. For instance, inactivation of the Hippo pathway or induction of the Dpp pathway in ISCs is sufficient to stimulate stem cell proliferation in the *Drosophila* midgut [42] [61]. Similarly, the MAPK pathway has been demonstrated to be critical in ISCs for division and differentiation downstream of EGFR [19]. However, the regulation of these pathways is not always identical between cell types: while the SAPK kinase cascade is strongly required cell-autonomously for ISC activity [13], its effect on *upd3* induction in ECs is only marginal. Along these lines, MAPKs act downstream of growth factor receptors in ISCs, while we found that Src kinases trigger their activation in ECs. We thus propose that a single regulatory network controls ISC proliferation both cell-autonomously and cell non-autonomously and that the two processes are linked by the secretion of cytokines and growth factors.

### Conclusions

Altogether, the results of our study illustrate key aspects of the regulation of cytokine expression by intestinal cells in the gut. We identify microbe-responsive enhancers in the promoter of *upd3* that act as stress sensors, thanks to the cooperative regulation by multiple pathways. Dpp, Hippo, Src, SAPK and MAPK pathways all converge on the transcriptional regulation of *upd3*, thus acting together as a genetic network dedicated to damage detection and response. Strikingly, this genetic network controls both proliferation in stem cells, as well as the expression of cytokines in ECs to subsequently induce ISC proliferation. This genetic regulatory network therefore links stem cell proliferation and cytokine production in one common molecular framework, and paves the way for future studies to decrypt the link between inflammation and cancer in the gut.

## Materials and methods

### Fly stocks and husbandry

*Drosophila* stocks were maintained at room temperature (~23°C) on standard fly medium (sucrose, cornmeal, yeast, and agar). Control lines: as controls for Gal4 driver experiments, we used the F1 progeny of the driver line crossed to wild-type stocks such as Canton-S (Cs) (BDSC: 64349), and background matched stocks such as *attp2* (BDSC: 36303) and *attp40* (BDSC: 36304). Gal4 Drivers: *Myo1A-Gal4*, *UAS-GFP*, *tub-Gal80*^*TS*^*; upd3-lacZ* (*Myo*^*TS*^, EC-specific), *Su(H)GBE-Gal4;UAS-GFP*, *tub-Gal80*^*TS*^ (*Su(H)*^*TS*^, EB-specific) [17]. Conditional *Gal4*^*TS*^ flies were obtained by crossing virgin females of the driver strain with males of the *UAS-transgene* line. For RNAi and overexpression experiments, F1 progenies (*driver > UAS-transgene*) were raised at 18°C until 3 days after emergence, to allow for full gut development. Flies were then switched to 29°C for a week to allow for maximum transgene expression and RNAi-mediated gene knockdown. UAS-transgene stocks: RNAi transgenic fly lines were obtained from Bloomington (TRiP lines), VDRC (Vienna) or NIG (Japan), as specified in S2 Table. *UAS-Atf3 3xHA* was obtained from FlyORF. *UAS-Src42A*, *UAS-Src42A*^*YF*^, *UAS-Src64B*, *UAS-Src64B*^*YF*^ were generously provided by professor Tian Xu [74]. *UAS-bsk*^*DN*^*; IF/CyO* (BDSC: 6409), *UAS-Src42A-IR* (NIG-FLY: 7873R-2), *UAS-Src42A-IR* (NIG-FLY: 7873R-3), *UAS-Src64B-IR* (VDRC: 35252), *UAS-Src64B-IR* (NIG-FLY: 7524R-1)/*CyO; MKRS/TM6B*, *UAS-Src42A*^*YF5382B*^, *sb/TM6B*, *UAS-Src64B*^*YF161*^; *sb/TM6B*, *yw;;Src64B*^*YU1332*^ (BDSC: 7342), *w-; IF/CyO; UAS-csk/TM6B*, *w-; UAS-cpb7/Cyo; MKRS/TM6B*, *w-; IF/CyO; UAS-cpa attB/TM6B* [75], *w-; UAS-cpa-IRC10* [75], *w-; IF/CyO; UAS-cpb-IR/TM6B* (VDRC: 46668), *w-;;; zyx*^*D41*^ [76], were generously provided by Florence Janody. *UAS-Apoliner* and *Tub-Apoliner* were both generously provided by Jean-Paul Vincent [53]. Reporter lines: *upd3*.*1-lacZ*, *esg-lacZ*, *Myo-lacZ*, [17]. A complete list of the TRiP UAS-RNAi lines used in the TF screen can be found in S2 Table. A list of the additional transgenic lines used in this report can be found in S5 Table.

### Generation of Upd3 enhancer trap lines

Overlapping fragments of ~1.5Kb were cloned in front of GFP, starting from 4.2Kb upstream of the *upd3* start site and ending 7.3Kb downstream of the gene. These sequence fragments were designated putative *upd3* enhancer regions A-R and cloned into T vector followed by pH-stinger [77] to create 21 enhancer trap GFP vectors. Each vector was used to generate at least two enhancer trap GFP fly lines (to account for insertion position effects), which were then screened for capacity to drive GFP expression in the adult midgut under both basal conditions as well as *Ecc15* infection. In addition, two reporter transgenes expressing NLS-GFP, fused to the Upd3 protein and driven under the control of the full *upd3* locus and endogenous promoter (from 4.2Kb upstream of the *upd3* start site, up to 7.3Kb downstream of the gene), as well as the same reporter with enhancer B and C sequence regions deleted, were created and inserted at the *attP2* insertion site.

### Bacterial cultures and oral infection

*Erwinia carotovora ssp*. *carotovora 15* (*Ecc15*) *and Pseudomonas entomophila (Pe)* are two Gram-negative bacteria, pathogenic to the *Drosophila* midgut when ingested [1]. Bacteria were maintained on standard LB agar plates and *Pe* was plated from glycerol stocks for each experiment. Bacteria were cultured in LB broth at 29°C for 16 hours. Oral infection was performed as previously described [12]: flies were starved in empty vials for 2 hours at 29°C, then moved to fly vials in which the standard food was completely covered by a filter paper disc containing 150μl of either 2.5% sucrose solution (control), or 5% sucrose solutions mixed in equal volume with OD_600_ = 200 bacterial pellet, or a solution of 500μg/ml of bleomycin or 6% DSS. Orally treated flies were incubated at 29°C until dissection.

### Generation of axenic flies

3 to 5 day old flies were transferred on fresh fruit juice agar plates. After 1 day of habituation, flies were allowed to lay eggs for 4–6 hours. Eggs were first suspended in 1X PBS, rinsed in 70% EtOH for 1 minute and dechorionated using 10% bleach for ~10min. Eggs were then transferred under a sterile flow hood and further rinsed 3 times with sterile ddH_2_O. The eggs were finally transferred into sterile fly vials with sterilized fly food. Flies were tested for presence of bacteria after each experiment, by plating homogenates on MRS agar plates.

### Immunohistochemistry and fluorescence imaging

After dissection, *Drosophila* midguts were fixed in 4% paraformaldehyde in 1X PBS for 45 to 90 minutes and successively washed 3 times with 0.1% TritonX in PBS. Guts to be immunostained were then incubated for an hour in blocking solution (1% bovine serum albumin, 1% normal donkey serum, and 0.1% Triton X-100 in PBS). Overnight primary antibody staining was performed at RT. Guts were washed 3 times with 0.1% TritonX in PBS and ≥2 hour secondary antibody staining was performed in PBS. Primary antibodies used: rabbit anti-pH3 (1:000, EMD Millipore), rabbit anti-β-Galactosidase (1:1000, MP Biomedicals), and mouse anti-Prospero (1:100, DSHB). Secondary antibodies used: donkey anti-rabbit-555 (1:2000, Thermo Fisher), donkey anti-mouse-488 (1:2000, Thermo Fisher), and donkey anti-mouse-647 (1:1000, Thermo Fisher). DNA was stained in 1:50,000 DAPI (Sigma-Aldrich) in PBS and 0.1% TritonX for 30min, and samples received a final three washes in PBS before mounting in antifade medium (Citifluor AF1). Imaging was performed on a Zeiss LSM 700 fluorescent/confocal inverted microscope.

### β-Galactosidase titration assay

*Myo-Gal4*^*TS*^*; Upd3-lacZ* driver/reporter flies were crossed to RNAi or overexpression lines and their adult progeny were induced at 29°C for seven days, then treated with either sucrose (control) or *Ecc15* for 16 hours. Five midguts were dissected for each sample and homogenized in 100μl Z-buffer (60mM Na_2_HPO_4_, 60mM NaH_2_PO_4_, 10mM KCl, 1mM MgSO_4_, 50mM β-mercaptoethanol, adjusted pH to 8 with NaOH). Homogenates were then centrifuged and 40μl of supernatant was mixed with 250μl of 0.35mg/ml ONPG (o-nitrophenol-β-D-galactoside) in Z-buffer solution in the wells of a 96-well plate. Absorbance was then measured at 420nm in a plate-reader (spectra max plus, Molecular Devices) every minute for one hour at 37°C. Because the amount of ONPG added to the reaction is sufficient to saturate the β-Gal in the samples, the reaction rate (absorbance vs time) is proportional to the quantity of β-Gal in each sample, and thus the maximum reaction rate (V_max_) was used as a measure of the relative β-Gal quantity in each sample. For each experiment, the average of three controls was used as a reference and relative *upd3-lacZ* activity was calculated (S2 Table). The three controls used were: progeny of *Myo-Gal4*^*TS*^*; upd3-lacZ* virgins crossed to either the wild type strain, *Canton-S* (Cs), or the controls “*attP2”* and “*attP40”*. The *attP2* and *attP40* lines are background controls for the TRiP *UAS-RNAi* stocks, while Cs is a standard, laboratory wild-type fly line. We used the variation in *upd3-lacZ* activity between the three controls (S5A and S5B Fig) to determine a confidence interval and select positive hits in the screen results (lower than 0.6 and higher than 1.4 upon infection, lower than 0.5 and higher than 1.6 in UC conditions). We further confirmed the significance of these results by the calculation of z-scores for each RNAi knockdown tested (S2 Table).

### Survival experiments

*Myo-Gal4*^*TS*^*; upd3-lacZ* driver/reporter flies were crossed to the *UAS-RNAi* lines and their progeny were raised at 18°C. At 3-days post eclosure, 20 adult females were shifted to 29°C, the temperature at which all survival experiments were done to allow constant expression of the RNAi constructs. Day seven post-induction was considered day 0 of the survival studies. The controls used were the F1 progeny of crosses between our driver and the wild-type stock Cs, as well as the background-matched lines “*attP2*” and “*attP40*”. To evaluate possible background or off-target effects, multiple RNAi lines were used for each gene and the survival of all parental lines alone was also monitored. Survival was recorded in unchallenged (UC) conditions, in which flies were kept on standard cornmeal medium, and upon constant exposure to *Ecc15* (flies were transferred to new tubes with fresh *Ecc15* every 3 days). Deaths were monitored daily and plotted using the GraphPad Prism 7.0c software. Results of survival experiments are aggregates of 3 to 9 biological replicates and error bars represent standard errors. LT50s were determined using PROBIT analysis in R.

### RT-qPCR

Total RNA was extracted from 15 to 20 female fly midguts following standard protocol with Trizol (Invitrogen). Reverse transcription (RT) was performed using the qScript cDNA synthesis kit (Quanta) and quantitative PCR with SsoAdvanced Universal SYBR Green Supermix (Bio-Rad) and a CFX96 TouchTM Real-Time PCR Detection System (Bio-Rad). Measured mRNA quantities were normalized to control *Rp49* (*RpL32*) mRNA values.

### Yeast one-hybrid

The *upd3-lacZ* sequence was cloned into 4 fragments fused to the *HIS3* reporter to generate baits further tested in yeast one-hybrid. *HIS3* encodes an imidazoleglycerol-phosphate dehydratase, that catalyzes histidine synthesis, and the inhibitor 3-amino-1,2,4-triazole (3AT) competitively inhibits this activity. The higher the level of 3AT in the medium is, the higher *HIS3* expression needs to be to insure yeast growth, thus testing the strength of transactivation of the *bait-HIS3* in response to multiple TFs. Prior to the TF/bait interaction test, a self-activation test was performed to assess whether natural *S*. *cerevisiae* TFs are sufficient to induce basal *HIS3* expression. This test was performed by measuring the growth of eight independently transformed yeasts for each bait on SC-His plates with varying concentrations of 3AT (0, 10, 20, 40, 60, 80mM). For each bait, a transformant yeast that can grow on SC-His medium, but is unable to grow on medium supplemented with 3AT was selected.

The yeast one-hybrid assay was performed as previously described [27,78,79]. Briefly, *upd3-HIS3* baits were integrated in the genome of *Saccharomyces cerevisiae* and transformed with a collection of 670 plasmids containing *Drosophila* TF open reading frames fused to the Gal4 activation domain. Each colony was plated on synthetic complete medium lacking Histidine (to select for the *upd3*-His construct) and Tryptophan (to select for the presence of the TF vector). Plates were incubated at 30°C for 3, 7, and 10d and imaged using a Bio-Rad gel doc system. Yeasts not transformed with any TF prey and yeasts transformed with the Gal4 activation domain alone served as negative control. Plate images were analyzed using the R package Gitter, that estimates colony surface and circularity. Sets of quadruplicate colonies that showed growth above background levels were deduced to have a direct interaction between the TF prey and the DNA bait, and the strength of the interaction was estimated and ranked (from +/- to +++) by the ability of each yeast colony to grow on increasing concentrations of the HIS3 inhibitor 3AT as previously described [27,79].

## Supporting information

S1 Fig Midgut *upd3* induction upon infection is controlled by multiple enhancer regions and is proportional to pathogen dose. (A, B) RT-qPCR measurements of *upd3* expression over multiple time points upon oral infection by *Ecc15* and *Pe*, respectively. Following either *Ecc15* or *Pe* infection, *upd3* induction peaks at 8-24h and returns to basal levels by 96h. (C) Enhancer regions G, H, K, P1 and P2 drive expression in discrete anatomical structures of the digestive tract. For a detailed description, see S1 Table. (D) Enhancers M and Q induce a constant signal in small epithelial cells. (E) Enhancer regions E and E0F seem to direct transcription inconsistently in a few scattered cells along the midgut upon infection. (F) Enhancer L drives GFP expression in salivary glands in response to infection. (G) *upd3* enhancer region B drives an infection-induced, EC-specific GFP signal, similar to that of enhancer region C. Mean values of at least 3 repeats are represented ± SEM. Scale bars are 50μm.
(TIF)

S2 Fig Qualitative comparison of visible *upd3-C-GFP* and *upd3-R-GFP* signals in CR and GF flies. (A, B) Enhancer regions C and R display no obvious difference in visible GFP signal between CR and GF conditions. Scale bars are 50μm.
(TIF)

S3 Fig Infection-induced *upd3* transcription occurs specifically in ECs and closely matches the *upd3-C-GFP* and *upd3-lacZ* signals. (A, B) RT-qPCR measurements of total gut *upd3* expression following EC (*Myo*) or EB (*Su(H)*)-specific knockdown of *upd3*, and *Ecc15* (A) or *Pe* (B) infection, indicates that most *upd3* induction is derived from ECs. (C) Accordingly, knockdown of *upd3* specifically in ECs (*Myo-Gal4*^*TS*^ driven UAS-RNAi) is adequate to strongly inhibit ISC proliferation in the midgut, as revealed by pH3+ cell counting. (D) A measure of GFP intensity in the cells of *upd3-C-GFP* guts for multiple time-points following *Ecc15* infection shows a peak in intensity at 8-24h and a return to basal levels by 96h. Black bars represent the median and blue diamonds represent the mean GFP intensity for each time point. (E) The signals driven by *upd3-C-GFP* and *upd3-lacZ* are induced upon *Ecc15* infection and overlap in the same ECs. White arrows indicate cells in which *upd3-C-GFP* and *upd3-lacZ* expression overlap. Statistical significance: mean values of at least 3 repeats are represented ± SE. *p<0.05, **p<0.01, ***p<0.001 (student’s t-test). Scale bar is 50μm.
(TIF)

S4 Fig Enhancer sequence B-C is critical for driving infection-induced expression of genes using the *upd3* locus. (A) Schematic of the *upd3* gene and the 21 overlapping sequences used to create GFP reporter lines. The *upd3* exons are represented by orange blocks and the introns are light blue. Putative enhancer regions have been color coded by their ability to drive GFP expression as follows: Solid Grey–no midgut signal, Green–infection-induced signal throughout the gut. (B) A sequence covering the *upd3* locus is capable of directing an infection-induced GFP signal in the midgut, but is unable to after the deletion of enhancer sequence B-C. Scale bars are 50μm.
(TIF)

S5 Fig The regulation of Upd3 differs in basal and infected conditions. (A-B) The relative *upd3-lacZ* values for each of the 718 lines used in our screen in either UC conditions (A) or upon infection (B) are depicted here. Three controls are used in these experiments (*Myo-Gal4*^*TS*^*; upd3-lacZ* x *attP2*, *attP40* and *Cs*) and their variation across 66 sets of experiments is depicted (brown, vertical line of points). The distribution of these control values due to inter-experimental variation was used to establish thresholds for determining positive hits (yellow dotted line is the threshold for increased expression and blue dotted line is the threshold for decreased expression). (C-D) Venn diagrams representing the overlap between TF hits inducing (C) or reducing (D) *upd3-lacZ* activity when knocked-down in ECs, in both basal condition and upon infection, showing only minor overlap between the two conditions. (E) Venn diagram showing the overlap between TFs considered as positive hits in our screen and their expression in ECs and/or regulation upon oral infection (based on [28]). Positive hits in the screen are enriched in genes that are expressed and regulated in ECs. (F) A scatter plot representing the relative effect of each TF on basal (x-axis) and infected (y-axis) conditions demonstrates that TFs modulating *upd3-lacZ* activity in UC and infected conditions are not correlated. Control samples are represented by orange points. (G) Gene Ontology Enrichment analysis demonstrates that the positive hit TFs identified in our screen are strongly enriched for involvement in development and epithelium morphogenesis as shown in this table. Statistical significance: mean values of at least 3 repeats are represented ± SE. *p<0.05, **p<0.01, ***p<0.001 (student’s t-test).
(TIF)

S6 Fig Knockdown of the Yki inhibitor, Hippo, is not sufficient to induce *upd3*. Basal *upd3* expression, as reported by *upd3-lacZ* activity, is not significantly induced by EC-specific knockdown of *hippo*. Statistical significance: mean values of at least 3 repeats are represented ± SE. *p<0.05, **p<0.01, ***p<0.001 (student’s t-test).
(TIF)

S7 Fig *upd3* expression in ECs is not dependent on apoptosis or autophagy. (A, B) Overexpression of caspases or autophagy genes is sufficient to induce *upd3* expression, as measured by *upd3-lacZ* (A). However, blocking apoptosis or autophagy by RNAi against caspases or autophagy genes, or overexpression of P35, does not impede *Ecc15*-induced *upd3* transcription (B). (C, D) The Apoliner construct expresses a membrane-bound mRFP fluorophore with a caspase sensitive site attached to an intracellular eGFP fluorophore. Caspase 3 (Cas3) cleaves this linker region, releasing the GFP fluorophore and allowing it to re-localize to the nucleus (C). *UAS-Apoliner*, driven by *NP1-Gal4*, marks apoptotic ECs with GFP localized to the nucleus (D). In *Ecc15* infected guts, we observe ECs that are caspase active and *upd3*-negative (white arrowhead), caspase inactive and *upd3*-positive (white circle), and both caspase-active and *upd3*-positive (white arrow). Scale bars are 25μm. Statistical significance: mean values of at least 3 repeats are represented ± SE. *p<0.05, **p<0.01, ***p<0.001 (student’s t-test).
(TIF)

S8 Fig Activation of JNK, Hep, Ras and major RTKs induce *upd3* expression but are not required for infection-induced expression. (A, B) Immunostaining against phosphorylated forms of JNKand p38 reveals that these kinases are activated in response to infection in ECs. (C) EC-specific inhibition of Bsk, Hep, or Ras, by RNAi or by expression of dominant negative forms has minimal effect on *Ecc15*-induced *upd3* expression. (D) EC-specific depletion of the MAPKKKs, MEKK1, ASK1, and TAK1 has no major effect on *Ecc15*-induced *upd3* expression. (E) Finally, EC-specific inhibition of EGFR, Pvr, or Pvf2 has no negative effect on *upd3-lacZ* activity, although activation of EGFR in ECs is sufficient to trigger *upd3-lacZ* expression. (F) Schematic of the SAPK/MAPK network. The AP-1 complex (D-Jun and D-Fos) is regulated by both Stress Activated Protein Kinase (SAPKs) and Mitogen Activated Protein Kinases (MAPKs). SAPKs lead to the activation of JNK, and MAPKs result in the activation of terminal kinases, including p38 and ERK. Statistical significance: mean values of at least 3 repeats are represented ± SE. *p<0.05, **p<0.01, ***p<0.001 (student’s t test).
(TIF)

S9 Fig Knockdown of positive regulators of *upd3* identified from screening reduces lifespan. (A-C) RNAi mediated knockdown of epigenetic regulators and homeobox genes (A), Hippo and Dpp pathway genes (B), or SAPK and MAPK constituents (C) reduces the lifespan of unchallenged flies. Curves represent averaged survival ± SE. *p<0.0332, **p<0.0021, ***p<0.0002, ****p<0.0001 (Log-rank test).
(TIF)

S10 Fig Model of Upd3 regulation in midgut ECs in response to enteric stress. Schematic representation of the pathways that control *upd3* transcription in ECs during intestinal trauma. Biotic and abiotic stresses, as well as the responsive ROS production, induce epithelial cell extrusion and cell death. The Sna TF may act as an integral component of cellular extrusion by negatively regulating cellular adhesion. SFK and MAPK pathways are activated by cellular stress, and converge on the activation of D-Fos and D-Jun TFs. The Hippo pathway likely responds to tissue loss in the midgut by removing the inhibition of the Yki and Sd TF complex.
(TIF)

S1 Table *upd3*-enhancer-GFP line summary. This table describes the precise expression pattern of our *upd3*-enhancer-GFP lines.
(XLSX)

S2 Table TF TRiP line summary from upd3-lacZ screen. This table describes the results of our functional screen. Relative lacZ activity values are indicated, as well as the presence of a TFBS for that transcription factor and the interaction in one hybrid screen (Y1H).
(XLSX)

S3 Table Yeast one-hybrid assay summary. This table describes the transcription factors found to bind upd3 by one hybrid. The score reflects the intensity of the binding which results in a stronger growth on selective medium (see Mat&Met).
(XLSX)

S4 Table LT50 Values of survival experiments. This table records the LT50 values and confidence intervals for the lines tested in survival experiments, under *Ecc15*-infected and unchallenged (UC) conditions. The first sub-table presents results associated with the main and supplementary figures (Fig 7B–7D, S9A–S9C Fig) and the additional sub-tables present the results of independent validation experiments, showing additionally tested RNAi lines for the same genes, as well as the survival data of parental lines. Blue text represents lines that are in the *attP2* background, green text represents those that are in the *attP40* background.
(XLSX)

S5 Table Additional *Drosophila* stocks. Lines used in this study and their provenance.
(XLSX)

## References

[1] Buchon N, Broderick NA, Lemaitre B. Gut homeostasis in a microbial world: insights from Drosophila melanogaster. Nat Rev Micro. 2013;11: 615–626. doi: 10.1038/nrmicro3074 23893105 10.1038/nrmicro3074

[2] Peterson LW, Artis D. Intestinal epithelial cells: regulators of barrier function and immune homeostasis. Nat Rev Immunol. 2014;14: 141–153. doi: 10.1038/nri3608 24566914 10.1038/nri3608

[3] Karin M, Clevers H. Reparative inflammation takes charge of tissue regeneration. Nature. 2016;529: 307–315. doi: 10.1038/nature17039 26791721 10.1038/nature17039 PMC5228603

[4] Radtke F, Clevers H. Self-renewal and cancer of the gut: two sides of a coin. Science. 2005;307: 1904–1909. doi: 10.1126/science.1104815 15790842 10.1126/science.1104815

[5] Bonfini A, Liu X, Buchon N. From pathogens to microbiota: How Drosophila intestinal stem cells react to gut microbes. Dev Comp Immunol. 2016;64: 22–38. doi: 10.1016/j.dci.2016.02.008 26855015 10.1016/j.dci.2016.02.008

[6] Apidianakis Y, Rahme L. Drosophila melanogaster as a model for human intestinal infection and pathology. Dis Model Mech. 2011;4: 21–30. doi: 10.1242/dmm.003970 21183483 10.1242/dmm.003970 PMC3014343

[7] Zeng X, Hou SX. Enteroendocrine cells are generated from stem cells through a distinct progenitor in the adult Drosophila posterior midgut. The Company of Biologists Limited; 2015;142: 644–653. doi: 10.1242/dev.113357 25670791 10.1242/dev.113357 PMC4325374

[8] Buchon N, Osman D, David FPA, Yu Fang H, Boquete J-P, Deplancke B, et al Morphological and molecular characterization of adult midgut compartmentalization in Drosophila. Cell Rep. 2013;3: 1725–1738. doi: 10.1016/j.celrep.2013.04.001 23643535 10.1016/j.celrep.2013.04.001

[9] Marianes A, Spradling AC, Brand A. Physiological and stem cell compartmentalization within the Drosophila midgut. elife. eLife Sciences Publications Limited; 2013;2 doi: 10.7554/eLife.00886 23991285 10.7554/eLife.00886 PMC3755342

[10] Ryu J-H, Ha E-M, Oh C-T, Seol J-H, Brey PT, Jin I, et al An essential complementary role of NF-kappaB pathway to microbicidal oxidants in Drosophila gut immunity. EMBO J. 2006;25: 3693–3701. doi: 10.1038/sj.emboj.7601233 16858400 10.1038/sj.emboj.7601233 PMC1538556

[11] Ha E-M, Lee K-A, Seo YY, Kim S-H, Lim J-H, Oh B-H, et al Coordination of multiple dual oxidase-regulatory pathways in responses to commensal and infectious microbes in drosophila gut. Nat Immunol. 2009;10: 949–957. doi: 10.1038/ni.1765 19668222 10.1038/ni.1765

[12] Buchon N, Broderick NA, Poidevin M, Pradervand S, Lemaitre B. Drosophila intestinal response to bacterial infection: activation of host defense and stem cell proliferation. Cell Host Microbe. 2009;5: 200–211. doi: 10.1016/j.chom.2009.01.003 19218090 10.1016/j.chom.2009.01.003

[13] Buchon N, Broderick NA, Chakrabarti S, Lemaitre B. Invasive and indigenous microbiota impact intestinal stem cell activity through multiple pathways in Drosophila. Genes Dev. Cold Spring Harbor Lab; 2009;23: 2333–2344. doi: 10.1101/gad.1827009 19797770 10.1101/gad.1827009 PMC2758745

[14] Bosco-Drayon V, Poidevin M, Boneca IG, Narbonne-Reveau K, Royet J, Charroux B. Peptidoglycan Sensing by the Receptor PGRP-LE in the Drosophila Gut Induces Immune Responses to Infectious Bacteria and Tolerance to Microbiota. Cell Host Microbe. 2012;12: 153–165. doi: 10.1016/j.chom.2012.06.002 22901536 10.1016/j.chom.2012.06.002

[15] Neyen C, Poidevin M, Roussel A, Lemaitre B. Tissue- and Ligand-Specific Sensing of Gram-Negative Infection in Drosophila by PGRP-LC Isoforms and PGRP-LE. J Immunol. 2012 doi: 10.4049/jimmunol.1201022 22772451 10.4049/jimmunol.1201022

[16] Osman D, Buchon N, Chakrabarti S, Huang Y-T, Su W-C, Poidevin M, et al Autocrine and paracrine unpaired signaling regulate intestinal stem cell maintenance and division. J Cell Sci. The Company of Biologists Ltd; 2012;125: 5944–5949. doi: 10.1242/jcs.113100 23038775 10.1242/jcs.113100

[17] Jiang H, Patel PH, Kohlmaier A, Grenley MO, Mcewen DG, Edgar BA. Cytokine/Jak/Stat signaling mediates regeneration and homeostasis in the Drosophila midgut. Cell. 2009;137: 1343–1355. doi: 10.1016/j.cell.2009.05.014 19563763 10.1016/j.cell.2009.05.014 PMC2753793

[18] Chatterjee M, Ip YT. Pathogenic stimulation of intestinal stem cell response in Drosophila. J Cell Physiol. 2009;220: 664–671. doi: 10.1002/jcp.21808 19452446 10.1002/jcp.21808 PMC4003914

[19] Buchon N, Broderick NA, Kuraishi T, Lemaitre B. Drosophila EGFR pathway coordinates stem cell proliferation and gut remodeling following infection. BMC Biol. BioMed Central Ltd; 2010;8: 152 doi: 10.1186/1741-7007-8-152 21176204 10.1186/1741-7007-8-152 PMC3022776

[20] Cordero JB, Stefanatos RK, Scopelliti A, Vidal M, Sansom OJ. Inducible progenitor-derived Wingless regulates adult midgut regeneration in Drosophila. EMBO J. 2012 doi: 10.1038/emboj.2012.248 22948071 10.1038/emboj.2012.248 PMC3463851

[21] Biteau B, Jasper H. EGF signaling regulates the proliferation of intestinal stem cells in Drosophila. 2011;138: 1045–1055. doi: 10.1242/dev.056671 21307097 10.1242/dev.056671 PMC3042864

[22] Jiang H, Grenley MO, Bravo M-J, Blumhagen RZ, Edgar BA. EGFR/Ras/MAPK signaling mediates adult midgut epithelial homeostasis and regeneration in Drosophila. Cell Stem Cell. 2011;8: 84–95. doi: 10.1016/j.stem.2010.11.026 21167805 10.1016/j.stem.2010.11.026 PMC3021119

[23] Zhou F, Rasmussen A, Lee S, Agaisse H. The UPD3 cytokine couples environmental challenge and intestinal stem cell division through modulation of JAK/STAT signaling in the stem cell microenvironment. Dev Biol. 2013;373: 383–393. doi: 10.1016/j.ydbio.2012.10.023 23110761 10.1016/j.ydbio.2012.10.023 PMC3534909

[24] Karpac J, Biteau B, Jasper H. Misregulation of an adaptive metabolic response contributes to the age-related disruption of lipid homeostasis in Drosophila. Cell Rep. 2013;4: 1250–1261. doi: 10.1016/j.celrep.2013.08.004 24035390 10.1016/j.celrep.2013.08.004 PMC3832190

[25] Amcheslavsky A, Jiang J, Ip YT. Tissue damage-induced intestinal stem cell division in Drosophila. Cell Stem Cell. 2009;4: 49–61. doi: 10.1016/j.stem.2008.10.016 19128792 10.1016/j.stem.2008.10.016 PMC2659574

[26] Broderick NA, Buchon N, Lemaitre B. Microbiota-induced changes in drosophila melanogaster host gene expression and gut morphology. MBio. American Society for Microbiology; 2014;5: e01117–14. doi: 10.1128/mBio.01117-14 24865556 10.1128/mBio.01117-14 PMC4045073

[27] Hens K, Feuz J-D, Isakova A, Iagovitina A, Massouras A, Bryois J, et al Automated protein-DNA interaction screening of Drosophila regulatory elements. Nat Meth. 2011 doi: 10.1038/nmeth.1763 22037703 10.1038/nmeth.1763 PMC3929264

[28] Dutta D, Dobson AJ, Houtz PL, Gläßer C, Revah J, Korzelius J, et al Regional Cell-Specific Transcriptome Mapping Reveals Regulatory Complexity in the Adult Drosophila Midgut. Cell Rep. 2015;12: 346–358. doi: 10.1016/j.celrep.2015.06.009 26146076 10.1016/j.celrep.2015.06.009

[29] Dutta D, Buchon N, Xiang J, Edgar BA. Regional Cell Specific RNA Expression Profiling of FACS Isolated Drosophila Intestinal Cell Populations. Curr Protoc Stem Cell Biol. Hoboken, NJ, USA: John Wiley & Sons, Inc; 2015;34: 2F.2.1–2F.2.14. doi: 10.1002/9780470151808.sc02f02s34 26237570 10.1002/9780470151808.sc02f02s34

[30] Mathelier A, Zhao X, Zhang AW, Parcy F, Worsley-Hunt R, Arenillas DJ, et al JASPAR 2014: an extensively expanded and updated open-access database of transcription factor binding profiles. Nucleic Acids Research. 2014;42: D142–7. doi: 10.1093/nar/gkt997 24194598 10.1093/nar/gkt997 PMC3965086

[31] Gallo SM, Gerrard DT, Miner D, Simich M, Soye Des B, Bergman CM, et al REDfly v3.0: toward a comprehensive database of transcriptional regulatory elements in Drosophila. Nucl Acids Res. 2010;39: D118–D123. doi: 10.1093/nar/gkq999 20965965 10.1093/nar/gkq999 PMC3013816

[32] Bothma JP, Magliocco J, Levine MS. The Snail Repressor Inhibits Release, not Elongation, of Paused Pol II in the Drosophila Embryo. Current biology: CB. NIH Public Access; 2011;21: 1571–1577. doi: 10.1016/j.cub.2011.08.019 21920753 10.1016/j.cub.2011.08.019 PMC3186076

[33] Chopra VS, Kong N, Levine MS. Transcriptional repression via antilooping in the Drosophila embryo. Proceedings of the National Academy of Sciences. National Acad Sciences; 2012;109: 9460–9464. doi: 10.1073/pnas.1102625108 22645339 10.1073/pnas.1102625108 PMC3386088

[34] Qi D, Bergman M, Aihara H, Nibu Y, Mannervik M. Drosophila Ebi mediates Snail-dependent transcriptional repression through HDAC3-induced histone deacetylation. EMBO J. European Molecular Biology Organization; 2008;27: 898–909. doi: 10.1038/emboj.2008.26 18309295 10.1038/emboj.2008.26 PMC2274935

[35] Nibu Y, Zhang H, Bajor E, Barolo S, Small S, Levine MS. dCtBP mediates transcriptional repression by Knirps, Krüppel and Snail in the Drosophila embryo. The EMBO Journal. European Molecular Biology Organization; 1998;17: 7009–7020. doi: 10.1093/emboj/17.23.7009 9843507 10.1093/emboj/17.23.7009 PMC1171049

[36] Huang J, Wu S, Barrera J, Matthews K, Pan D. The Hippo signaling pathway coordinately regulates cell proliferation and apoptosis by inactivating Yorkie, the Drosophila Homolog of YAP. Cell. 2005;122: 421–434. doi: 10.1016/j.cell.2005.06.007 16096061 10.1016/j.cell.2005.06.007

[37] Staley BK, Irvine KD. Hippo signaling in Drosophila: Recent advances and insights. Singh A, Irvine KD, editors. Dev Dyn. 2011;241: 3–15. doi: 10.1002/dvdy.22723 22174083 10.1002/dvdy.22723 PMC3426292

[38] Li Q, Li S, Mana-Capelli S, Roth Flach RJ, Danai LV, Amcheslavsky A, et al The Conserved Misshapen-Warts-Yorkie Pathway Acts in Enteroblasts to Regulate Intestinal Stem Cells in Drosophila. Dev Cell. 2014;31: 291–304. doi: 10.1016/j.devcel.2014.09.012 25453828 10.1016/j.devcel.2014.09.012 PMC4254555

[39] Qing Y, Yin F, Wang W, Zheng Y, Guo P, Schozer F, et al The Hippo effector Yorkie activates transcription by interacting with a histone methyltransferase complex through Ncoa6. elife. 2014;3: 1260 doi: 10.7554/eLife.02564 25027438 10.7554/eLife.02564 PMC4118621

[40] Bayarmagnai B, Nicolay BN, Islam ABMMK, Lopez-Bigas N, Frolov MV. Drosophila GAGA factor is required for full activation of the dE2f1-Yki/Sd transcriptional program. Cell Cycle. 2012;11: 4191–4202. doi: 10.4161/cc.22486 23070566 10.4161/cc.22486 PMC3524215

[41] Shaw RL, Kohlmaier A, Polesello C, Veelken C, Edgar BA, Tapon N. The Hippo pathway regulates intestinal stem cell proliferation during Drosophila adult midgut regeneration. 2010;137: 4147–4158. doi: 10.1242/dev.052506 21068063 10.1242/dev.052506 PMC2990206

[42] Staley BK, Irvine KD. Warts and Yorkie mediate intestinal regeneration by influencing stem cell proliferation. Curr Biol. 2010;20: 1580–1587. doi: 10.1016/j.cub.2010.07.041 20727758 10.1016/j.cub.2010.07.041 PMC2955330

[43] Guo Z, Driver I, Ohlstein B. Injury-induced BMP signaling negatively regulates Drosophila midgut homeostasis. J Cell Biol. 2013;201: 945–961. doi: 10.1083/jcb.201302049 23733344 10.1083/jcb.201302049 PMC3678160

[44] Tian A, Jiang J. Intestinal epithelium-derived BMP controls stem cell self-renewal in Drosophila adult midgut. 2014;3: e01857 doi: 10.7554/eLife.01857 24618900 10.7554/eLife.01857 PMC3948108

[45] Zhou J, Florescu S, Boettcher A-L, Luo L, Dutta D, Kerr G, et al Dpp/Gbb signaling is required for normal intestinal regeneration during infection. Dev Biol. 2014 doi: 10.1016/j.ydbio.2014.12.017 25553980 10.1016/j.ydbio.2014.12.017

[46] Li H, Qi Y, Jasper H. Dpp signaling determines regional stem cell identity in the regenerating adult Drosophila gastrointestinal tract. Cell Rep. 2013;4: 10–18. doi: 10.1016/j.celrep.2013.05.040 23810561 10.1016/j.celrep.2013.05.040 PMC3778028

[47] Driver I, Ohlstein B. Specification of regional intestinal stem cell identity during Drosophila metamorphosis. 2014;141: 1848–1856. doi: 10.1242/dev.104018 24700821 10.1242/dev.104018 PMC3994771

[48] Li Z, Zhang Y, Han L, Shi L, Lin X. Trachea-derived dpp controls adult midgut homeostasis in Drosophila. Dev Cell. 2013;24: 133–143. doi: 10.1016/j.devcel.2012.12.010 23369712 10.1016/j.devcel.2012.12.010

[49] Aleman A, Rios M, Juarez M, Lee D, Chen A, Eivers E. Mad linker phosphorylations control the intensity and range of the BMP-activity gradient in developing Drosophila tissues. Sci Rep. 2014;4: 6927 doi: 10.1038/srep06927 25377173 10.1038/srep06927 PMC4223678

[50] Wisotzkey RG, Mehra A, Sutherland DJ, Dobens LL, Liu X, Dohrmann C, et al Medea is a Drosophila Smad4 homolog that is differentially required to potentiate DPP responses. Development. 1998;125: 1433–1445. 9502724 10.1242/dev.125.8.1433

[51] Kockel L, Homsy JG, Bohmann D. Drosophila AP-1: lessons from an invertebrate. Oncogene. 2001;20: 2347–2364. doi: 10.1038/sj.onc.1204300 11402332 10.1038/sj.onc.1204300

[52] Kappelmann M, Bosserhoff A, Kuphal S. AP-1/c-Jun transcription factors: regulation and function in malignant melanoma. Eur J Cell Biol. 2014;93: 76–81. doi: 10.1016/j.ejcb.2013.10.003 24315690 10.1016/j.ejcb.2013.10.003

[53] Bardet P-L, Kolahgar G, Mynett A, Miguel-Aliaga I, Briscoe J, Meier P, et al A fluorescent reporter of caspase activity for live imaging. Proceedings of the National Academy of Sciences. National Acad Sciences; 2008;105: 13901–13905. doi: 10.1073/pnas.0806983105 18779587 10.1073/pnas.0806983105 PMC2544551

[54] Chakrabarti S, Poidevin M, Lemaitre B. The Drosophila MAPK p38c Regulates Oxidative Stress and Lipid Homeostasis in the Intestine. Garsin DA, editor. PLoS Genet. Public Library of Science; 2014;10: e1004659 doi: 10.1371/journal.pgen.1004659 25254641 10.1371/journal.pgen.1004659 PMC4177744

[55] Stokoe D, McCormick F. Activation of c-Raf-1 by Ras and Src through different mechanisms: activation in vivo and in vitro. EMBO J. 1997;16: 2384–2396. doi: 10.1093/emboj/16.9.2384 9171352 10.1093/emboj/16.9.2384 PMC1169839

[56] Tran NH, Frost JA. Phosphorylation of Raf-1 by p21-activated kinase 1 and Src regulates Raf-1 autoinhibition. J Biol Chem. American Society for Biochemistry and Molecular Biology; 2003;278: 11221–11226. doi: 10.1074/jbc.M210318200 12551923 10.1074/jbc.M210318200

[57] Li H, Qi Y, Jasper H. Preventing Age-Related Decline of Gut Compartmentalization Limits Microbiota Dysbiosis and Extends Lifespan. Cell Host Microbe. 2016;19: 240–253. doi: 10.1016/j.chom.2016.01.008 26867182 10.1016/j.chom.2016.01.008 PMC5106289

[58] Buchon N, Silverman N, Cherry S. Immunity in Drosophila melanogaster—from microbial recognition to whole-organism physiology. Nat Rev Immunol. 2014;14: 796–810. doi: 10.1038/nri3763 25421701 10.1038/nri3763 PMC6190593

[59] Ha E-M, Lee K-A, Park SH, Kim S-H, Nam H-J, Lee H-Y, et al Regulation of DUOX by the Galphaq-phospholipase Cbeta-Ca2+ pathway in Drosophila gut immunity. Dev Cell. 2009;16: 386–397. doi: 10.1016/j.devcel.2008.12.015 19289084 10.1016/j.devcel.2008.12.015

[60] Jones RM, Luo L, Ardita CS, Richardson AN, Kwon YM, Mercante JW, et al Symbiotic lactobacilli stimulate gut epithelial proliferation via Nox-mediated generation of reactive oxygen species. EMBO J. 2013;32: 3017–3028. doi: 10.1038/emboj.2013.224 24141879 10.1038/emboj.2013.224 PMC3844951

[61] Ayyaz A, Li H, Jasper H. Haemocytes control stem cell activity in the Drosophila intestine. Nat Cell Biol. 2015;17: 736–748. doi: 10.1038/ncb3174 26005834 10.1038/ncb3174 PMC4449816

[62] Karpowicz P, Perez J, Perrimon N. The Hippo tumor suppressor pathway regulates intestinal stem cell regeneration. 2010;137: 4135–4145. doi: 10.1242/dev.060483 21098564 10.1242/dev.060483 PMC2990205

[63] Ren F, Wang B, Yue T, Yun E-Y, Ip YT, Jiang J. Hippo signaling regulates Drosophila intestine stem cell proliferation through multiple pathways. Proceedings of the National Academy of Sciences. 2010;107: 21064–21069. doi: 10.1073/pnas.1012759107 21078993 10.1073/pnas.1012759107 PMC3000252

[64] Li Q, Li S, Mana-Capelli S, Roth Flach RJ, Danai LV, Amcheslavsky A, et al The Conserved Misshapen-Warts-Yorkie Pathway Acts in Enteroblasts to Regulate Intestinal Stem Cells in Drosophila. Dev Cell. 2014;31: 291–304. doi: 10.1016/j.devcel.2014.09.012 25453828 10.1016/j.devcel.2014.09.012 PMC4254555

[65] Qiao Y, He H, Jonsson P, Sinha I, Zhao C, Dahlman-Wright K. AP-1 is a key regulator of proinflammatory cytokine TNFα-mediated triple-negative breast cancer progression. Journal of Biological Chemistry. American Society for Biochemistry and Molecular Biology; 2016;291: 18309–18309. doi: 10.1074/jbc.A115.702571 27566813 10.1074/jbc.A115.702571 PMC5000078

[66] Khalaf H, Jass J, Olsson P-E. Differential cytokine regulation by NF-kappaB and AP-1 in Jurkat T-cells. BMC Immunol. BioMed Central; 2010;11: 26 doi: 10.1186/1471-2172-11-26 20507572 10.1186/1471-2172-11-26 PMC2889865

[67] Yoo SK, Freisinger CM, LeBert DC, Huttenlocher A. Early redox, Src family kinase, and calcium signaling integrate wound responses and tissue regeneration in zebrafish. J Cell Biol. Rockefeller University Press; 2012;199: 225–234. doi: 10.1083/jcb.201203154 23045550 10.1083/jcb.201203154 PMC3471241

[68] Juarez MT, Patterson RA, Sandoval-Guillen E, McGinnis W. Duox, Flotillin-2, and Src42A Are Required to Activate or Delimit the Spread of the Transcriptional Response to Epidermal Wounds in Drosophila. PLoS Genet. 2011;7: e1002424 doi: 10.1371/journal.pgen.1002424 22242003 10.1371/journal.pgen.1002424 PMC3248467

[69] Sun G, Irvine KD. Regulation of Hippo signaling by Jun kinase signaling during compensatory cell proliferation and regeneration, and in neoplastic tumors. Dev Biol. 2011;350: 139–151. doi: 10.1016/j.ydbio.2010.11.036 21145886 10.1016/j.ydbio.2010.11.036 PMC3038240

[70] Taniguchi K, Wu L-W, Grivennikov SI, de Jong PR, Lian I, Yu F-X, et al A gp130–Src–YAP module links inflammation to epithelial regeneration. Nature. 2015;519: 57–62. doi: 10.1038/nature14228 25731159 10.1038/nature14228 PMC4447318

[71] Loudhaief R, Brun-Barale A, Benguettat O, Nawrot-Esposito M-P, Pauron D, Amichot M, et al Apoptosis restores cellular density by eliminating a physiologically or genetically induced excess of enterocytes in the Drosophila midgut. Development. Oxford University Press for The Company of Biologists Limited; 2017;144: 808–819. doi: 10.1242/dev.142539 28246211 10.1242/dev.142539

[72] Patel PH, Dutta D, Edgar BA. Niche appropriation by Drosophila intestinal stem cell tumours. Nat Cell Biol. 2015;17: 1182–1192. doi: 10.1038/ncb3214 26237646 10.1038/ncb3214 PMC4709566

[73] Cano A, Pérez-Moreno MA, Rodrigo I, Locascio A, Blanco MJ, del Barrio MG, et al The transcription factor Snail controls epithelial|[ndash]|mesenchymal transitions by repressing E-cadherin expression. Nat Cell Biol. Nature Publishing Group; 2000;2: 76–83. doi: 10.1038/35000025 10655586 10.1038/35000025

[74] Pedraza LG, Stewart RA, Li D-M, Xu T. Drosophila Src-family kinases function with Csk to regulate cell proliferation and apoptosis. Oncogene. Nature Publishing Group; 2004;23: 4754–4762. doi: 10.1038/sj.onc.1207635 15107833 10.1038/sj.onc.1207635

[75] Fernández BG, Jezowska B, Janody F. Drosophila actin-Capping Protein limits JNK activation by the Src proto-oncogene. Oncogene. 2013;33: 2027–2039. doi: 10.1038/onc.2013.155 23644660 10.1038/onc.2013.155

[76] Gaspar P, Holder MV, Aerne BL, Janody F, Tapon N. Zyxin antagonizes the FERM protein expanded to couple F-actin and Yorkie-dependent organ growth.—PubMed—NCBI. Current Biology. 2015;25: 679–689. doi: 10.1016/j.cub.2015.01.010 25728696 10.1016/j.cub.2015.01.010

[77] Barolo S, Carver LA, Posakony JW. GFP and beta-galactosidase transformation vectors for promoter/enhancer analysis in Drosophila. BioTechniques. 2000;29: 726–728–730–732. 11056799 10.2144/00294bm10

[78] Hens K, Feuz J-D, Deplancke B. A High-throughput Gateway-Compatible Yeast One-Hybrid Screen to Detect Protein-DNA Interactions. Methods Mol Biol. 2012;786: 335–355. doi: 10.1007/978-1-61779-292-2_20 21938636 10.1007/978-1-61779-292-2_20

[79] Kalay G, Lusk R, Dome M, Hens K, Deplancke B, Wittkopp PJ. Potential Direct Regulators of the Drosophila yellow Gene Identified by Yeast One-Hybrid and RNAi Screens. G3 (Bethesda). Genetics Society of America; 2016;6: 3419–3430. doi: 10.1534/g3.116.032607 27527791 10.1534/g3.116.032607 PMC5068961

