# Supplementary figures and images for: Hippo, TGF-β, and Src-MAPK pathways regulate transcription of the upd3 cytokine in Drosophila enterocytes upon bacterial infection
Source: PLoS Genet. 2017 Nov 6;13(11):e1007091. doi: 10.1371/journal.pgen.1007091 (PMC5690694; doi:10.1371/journal.pgen.1007091)

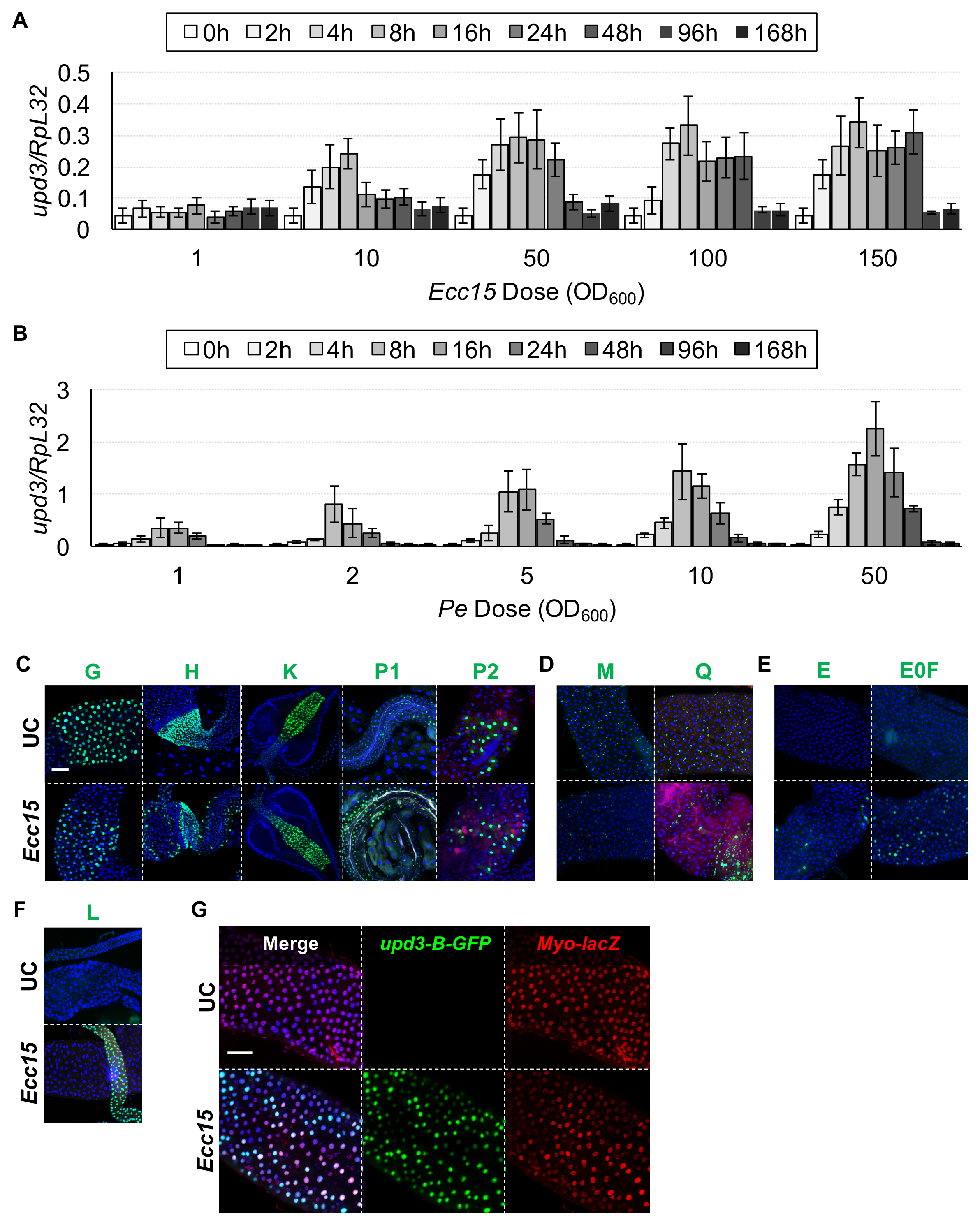

Supplement: S1 Fig — (A, B) RT-qPCR measurements of upd3 expression over multiple time points upon oral infection by Ecc15 and Pe, respectively. Following either Ecc15 or Pe infection, upd3 induction peaks at 8-24h and returns to basal levels by 96h. (C) Enhancer regions G, H, K, P1 and P2 drive expression in discrete anatomical structures of the digestive tract. For a detailed description, see S1 Table. (D) Enhancers M and Q induce a constant signal in small epithelial cells. (E) Enhancer regions E and E0F seem to direct transcription inconsistently in a few scattered cells along the midgut upon infection. (F) Enhancer L drives GFP expression in salivary glands in response to infection. (G) upd3 enhancer region B drives an infection-induced, EC-specific GFP signal, similar to that of enhancer region C. Mean values of at least 3 repeats are represented ± SEM. Scale bars are 50μm. (TIF) [file pgen.1007091.s001.tif]

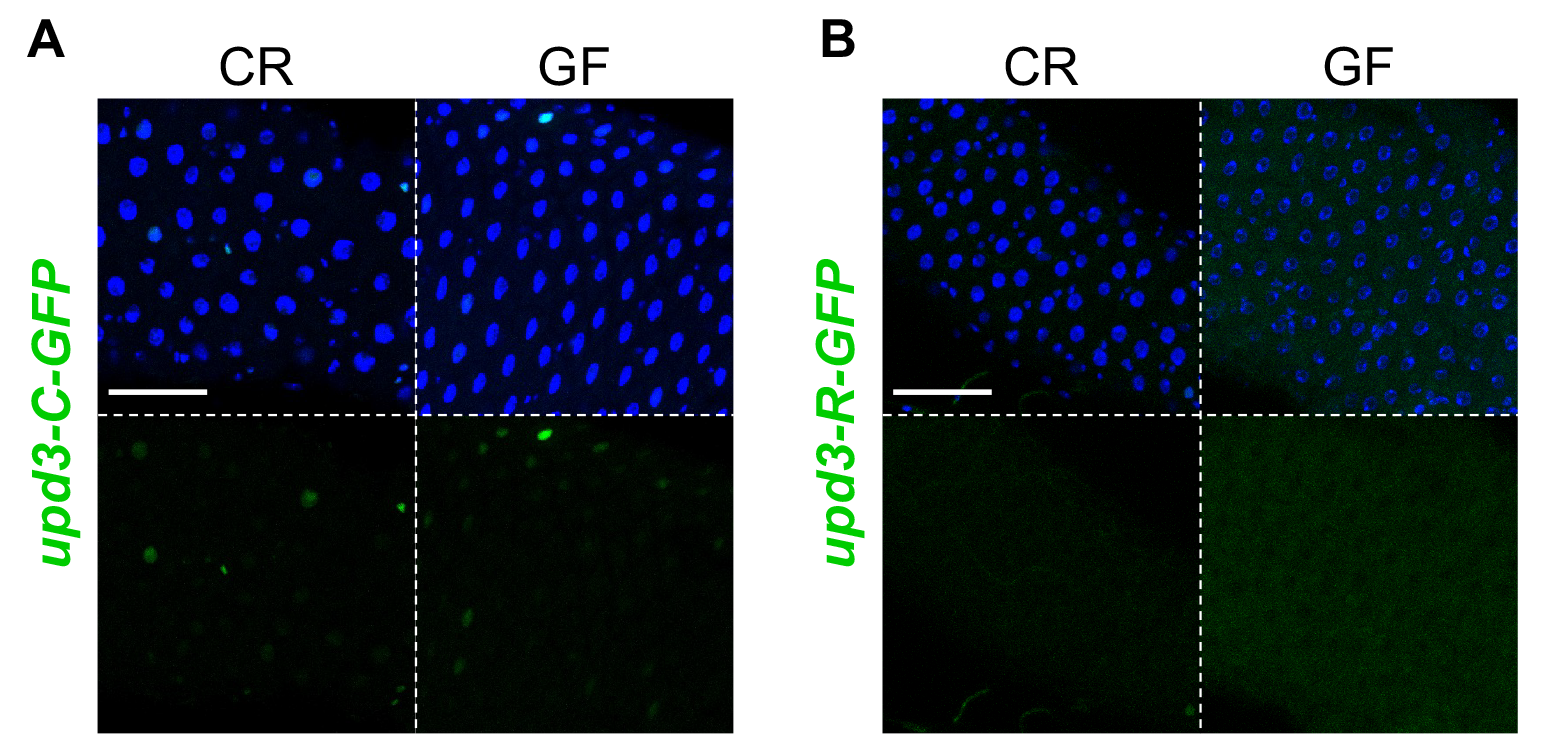

Supplement: S2 Fig — (A, B) Enhancer regions C and R display no obvious difference in visible GFP signal between CR and GF conditions. Scale bars are 50μm. (TIF) [file pgen.1007091.s002.tif]

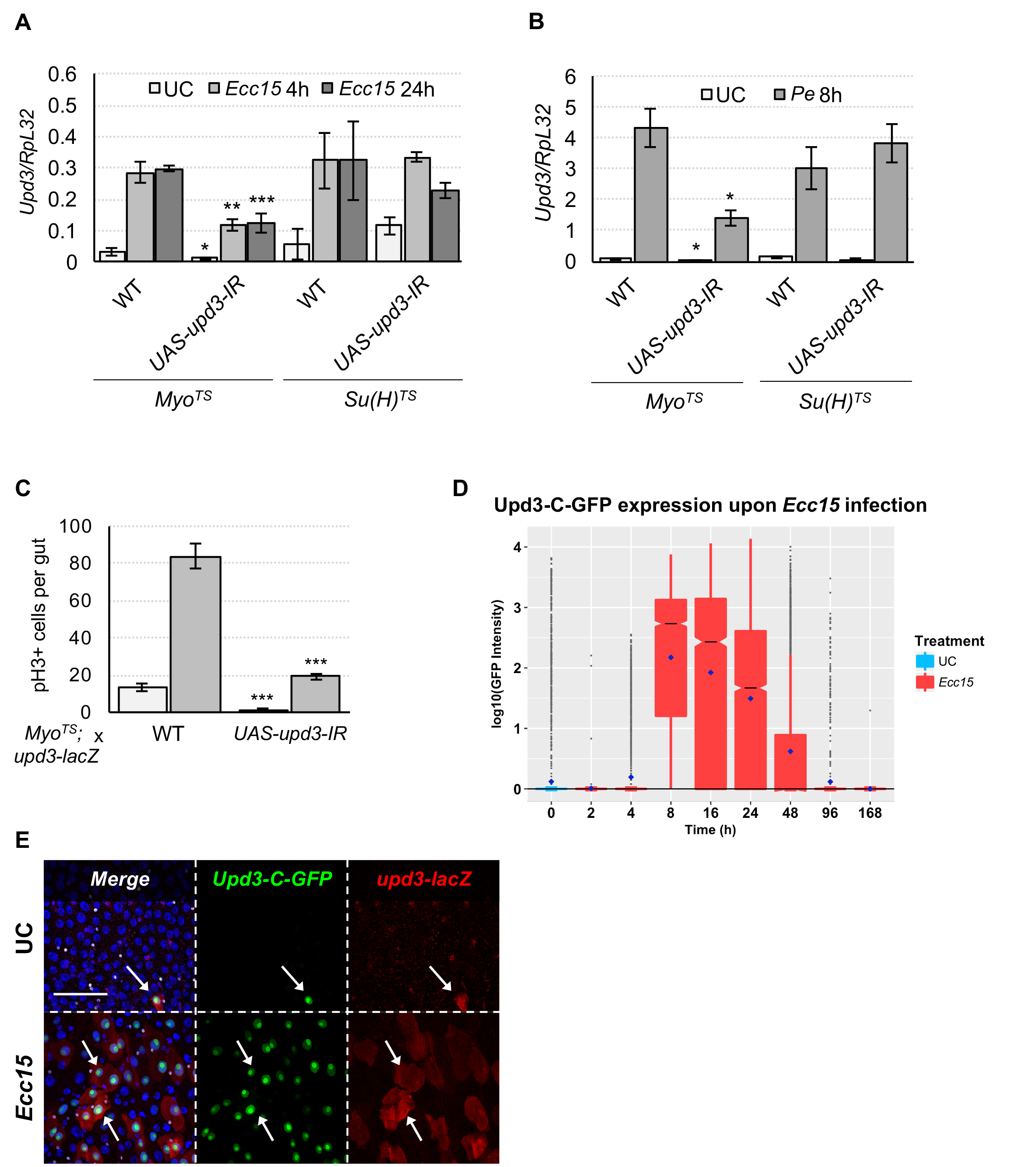

Supplement: S3 Fig — (A, B) RT-qPCR measurements of total gut upd3 expression following EC (Myo) or EB (Su(H))-specific knockdown of upd3, and Ecc15 (A) or Pe (B) infection, indicates that most upd3 induction is derived from ECs. (C) Accordingly, knockdown of upd3 specifically in ECs (Myo-Gal4TS driven UAS-RNAi) is adequate to strongly inhibit ISC proliferation in the midgut, as revealed by pH3+ cell counting. (D) A measure of GFP intensity in the cells of upd3-C-GFP guts for multiple time-points following Ecc15 infection shows a peak in intensity at 8-24h and a return to basal levels by 96h. Black bars represent the median and blue diamonds represent the mean GFP intensity for each time point. (E) The signals driven by upd3-C-GFP and upd3-lacZ are induced upon Ecc15 infection and overlap in the same ECs. White arrows indicate cells in which upd3-C-GFP and upd3-lacZ expression overlap. Statistical significance: mean values of at least 3 repeats are represented ± SE. *p<0.05, **p<0.01, ***p<0.001 (student’s t-test). Scale bar is 50μm. (TIF) [file pgen.1007091.s003.tif]

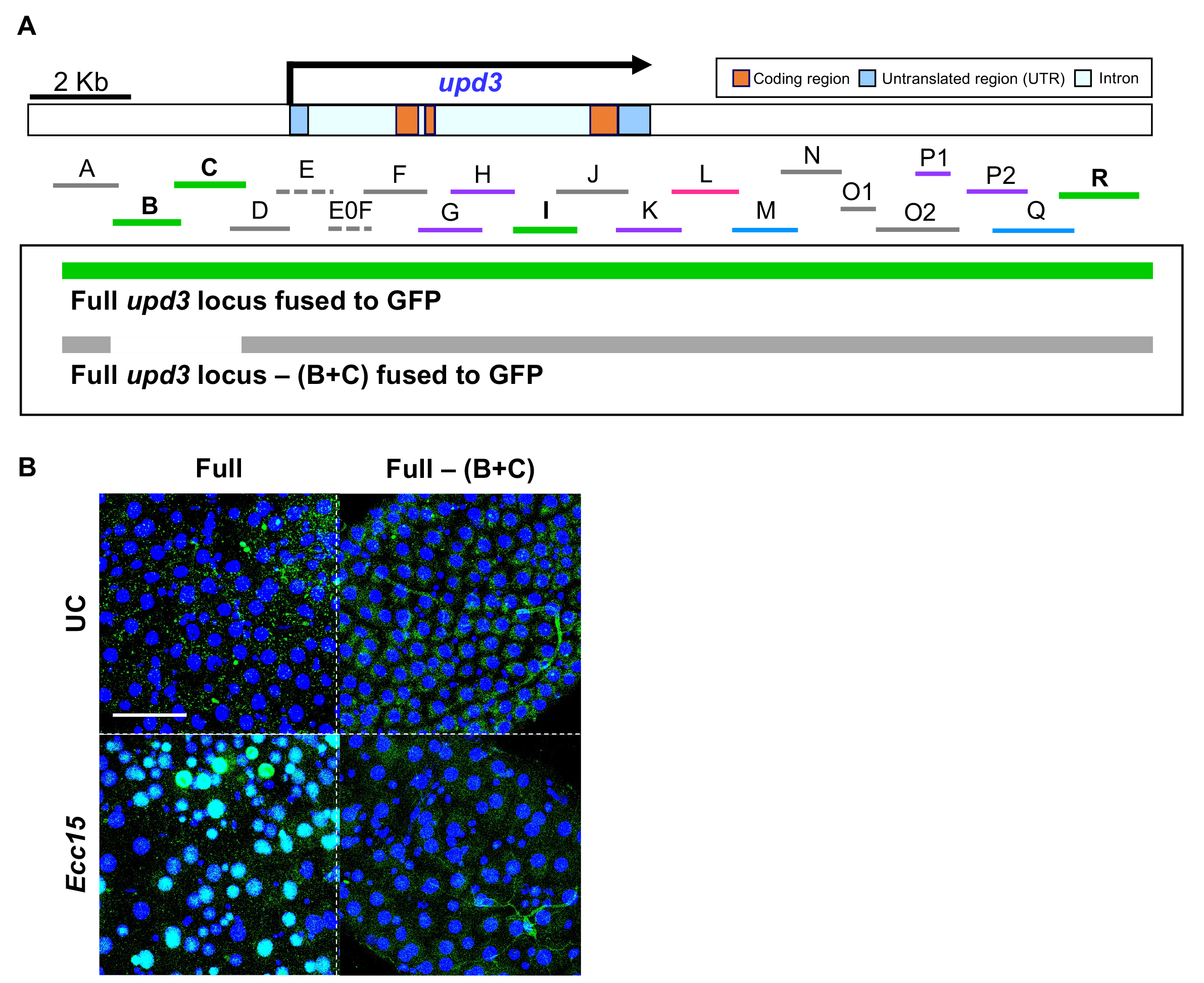

Supplement: S4 Fig — (A) Schematic of the upd3 gene and the 21 overlapping sequences used to create GFP reporter lines. The upd3 exons are represented by orange blocks and the introns are light blue. Putative enhancer regions have been color coded by their ability to drive GFP expression as follows: Solid Grey–no midgut signal, Green–infection-induced signal throughout the gut. (B) A sequence covering the upd3 locus is capable of directing an infection-induced GFP signal in the midgut, but is unable to after the deletion of enhancer sequence B-C. Scale bars are 50μm. (TIF) [file pgen.1007091.s004.tif]

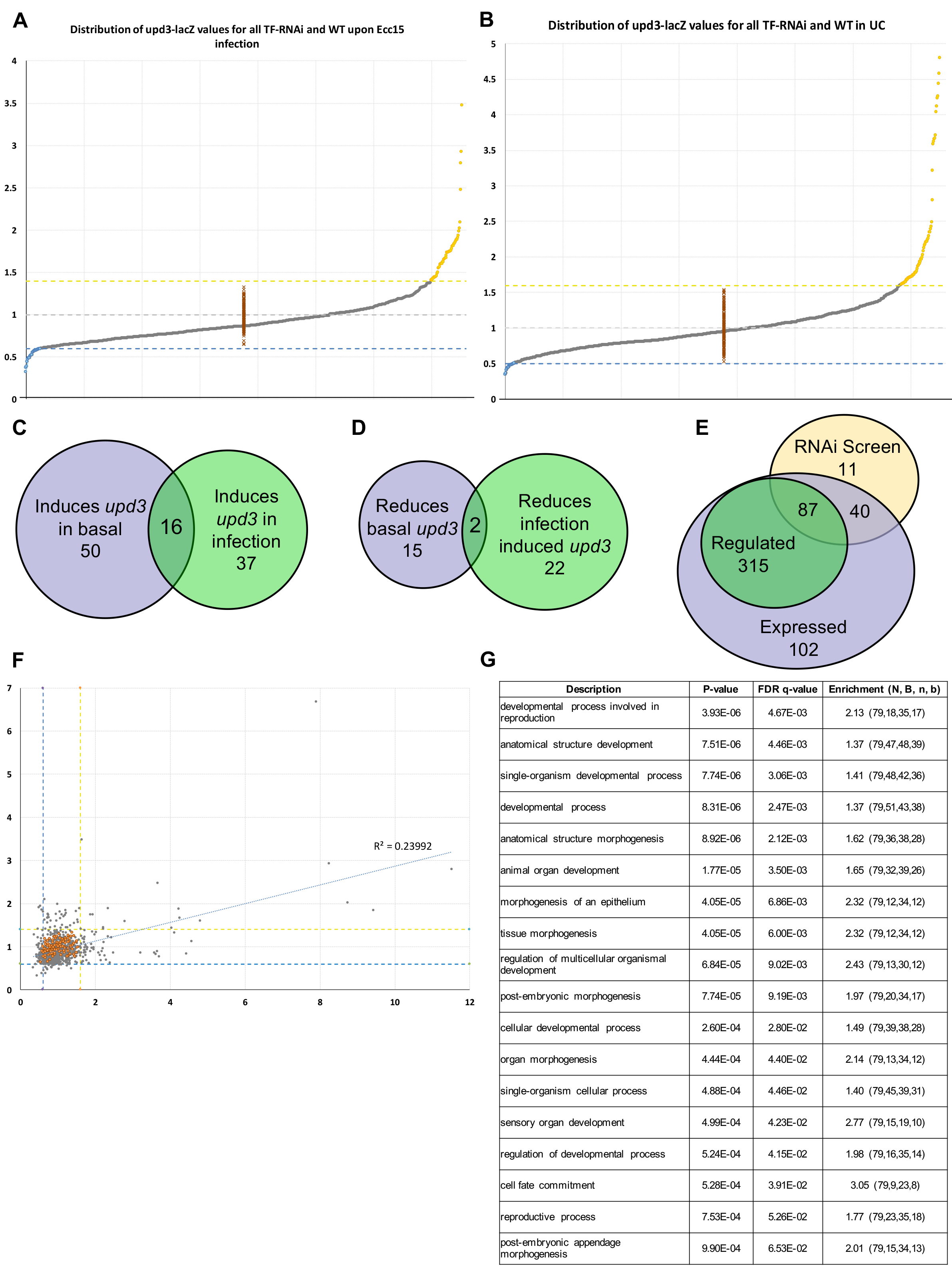

Supplement: S5 Fig — (A-B) The relative upd3-lacZ values for each of the 718 lines used in our screen in either UC conditions (A) or upon infection (B) are depicted here. Three controls are used in these experiments (Myo-Gal4TS; upd3-lacZ x attP2, attP40 and Cs) and their variation across 66 sets of experiments is depicted (brown, vertical line of points). The distribution of these control values due to inter-experimental variation was used to establish thresholds for determining positive hits (yellow dotted line is the threshold for increased expression and blue dotted line is the threshold for decreased expression). (C-D) Venn diagrams representing the overlap between TF hits inducing (C) or reducing (D) upd3-lacZ activity when knocked-down in ECs, in both basal condition and upon infection, showing only minor overlap between the two conditions. (E) Venn diagram showing the overlap between TFs considered as positive hits in our screen and their expression in ECs and/or regulation upon oral infection (based on [28]). Positive hits in the screen are enriched in genes that are expressed and regulated in ECs. (F) A scatter plot representing the relative effect of each TF on basal (x-axis) and infected (y-axis) conditions demonstrates that TFs modulating upd3-lacZ activity in UC and infected conditions are not correlated. Control samples are represented by orange points. (G) Gene Ontology Enrichment analysis demonstrates that the positive hit TFs identified in our screen are strongly enriched for involvement in development and epithelium morphogenesis as shown in this table. Statistical significance: mean values of at least 3 repeats are represented ± SE. *p<0.05, **p<0.01, ***p<0.001 (student’s t-test). (TIF) [file pgen.1007091.s005.tif]

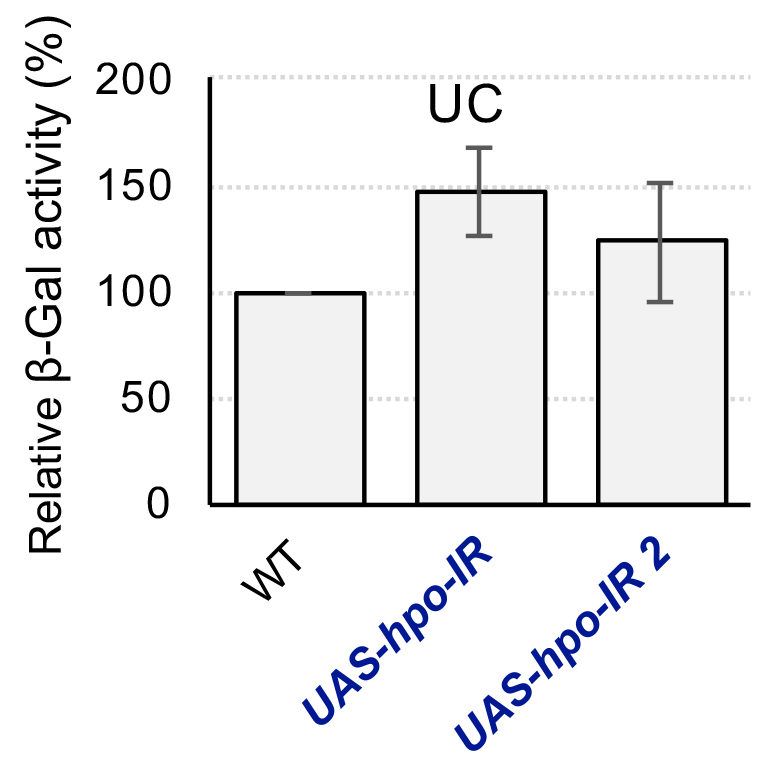

Supplement: S6 Fig — Basal upd3 expression, as reported by upd3-lacZ activity, is not significantly induced by EC-specific knockdown of hippo. Statistical significance: mean values of at least 3 repeats are represented ± SE. *p<0.05, **p<0.01, ***p<0.001 (student’s t-test). (TIF) [file pgen.1007091.s006.tif]

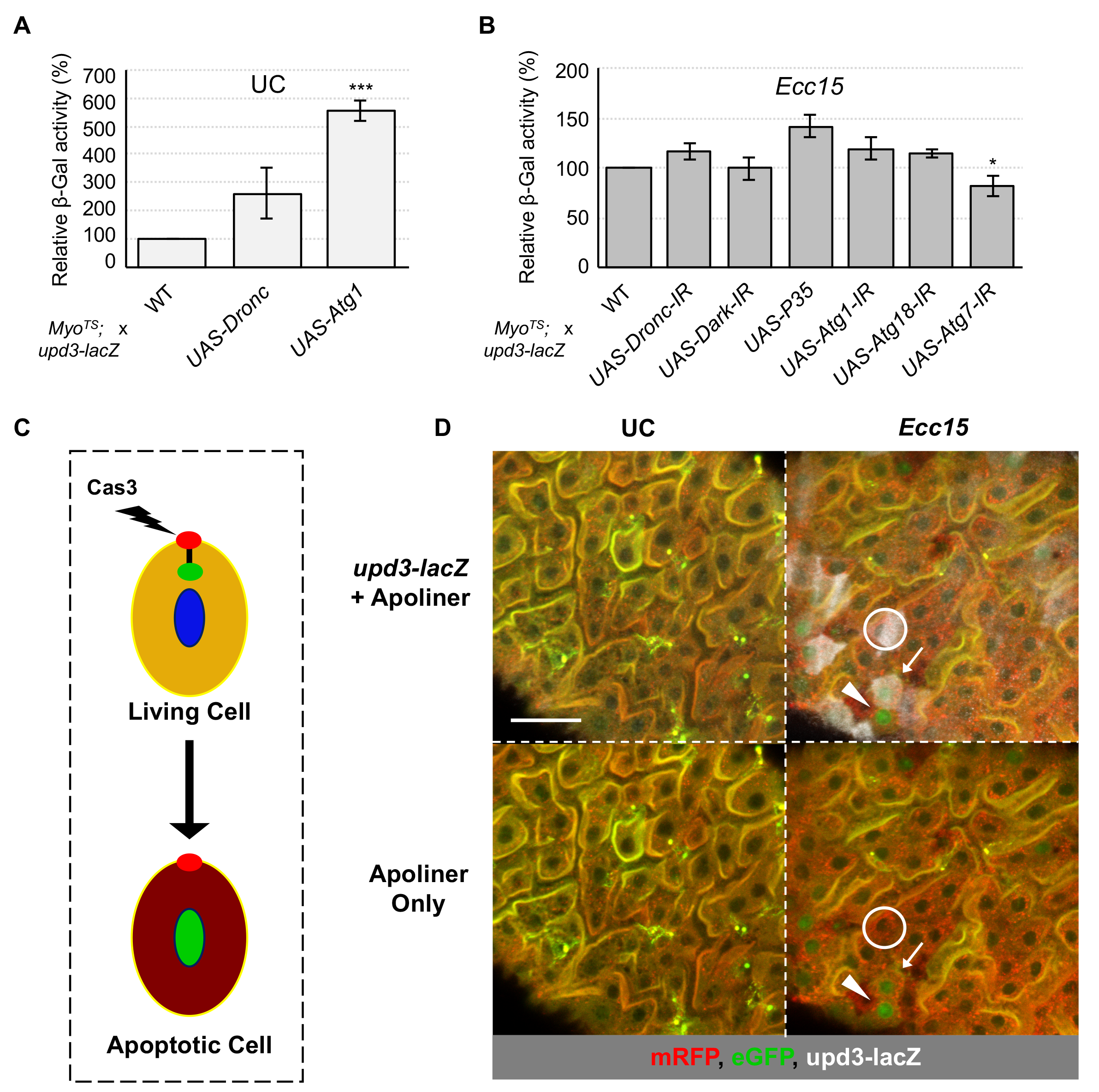

Supplement: S7 Fig — (A, B) Overexpression of caspases or autophagy genes is sufficient to induce upd3 expression, as measured by upd3-lacZ (A). However, blocking apoptosis or autophagy by RNAi against caspases or autophagy genes, or overexpression of P35, does not impede Ecc15-induced upd3 transcription (B). (C, D) The Apoliner construct expresses a membrane-bound mRFP fluorophore with a caspase sensitive site attached to an intracellular eGFP fluorophore. Caspase 3 (Cas3) cleaves this linker region, releasing the GFP fluorophore and allowing it to re-localize to the nucleus (C). UAS-Apoliner, driven by NP1-Gal4, marks apoptotic ECs with GFP localized to the nucleus (D). In Ecc15 infected guts, we observe ECs that are caspase active and upd3-negative (white arrowhead), caspase inactive and upd3-positive (white circle), and both caspase-active and upd3-positive (white arrow). Scale bars are 25μm. Statistical significance: mean values of at least 3 repeats are represented ± SE. *p<0.05, **p<0.01, ***p<0.001 (student’s t-test). (TIF) [file pgen.1007091.s007.tif]

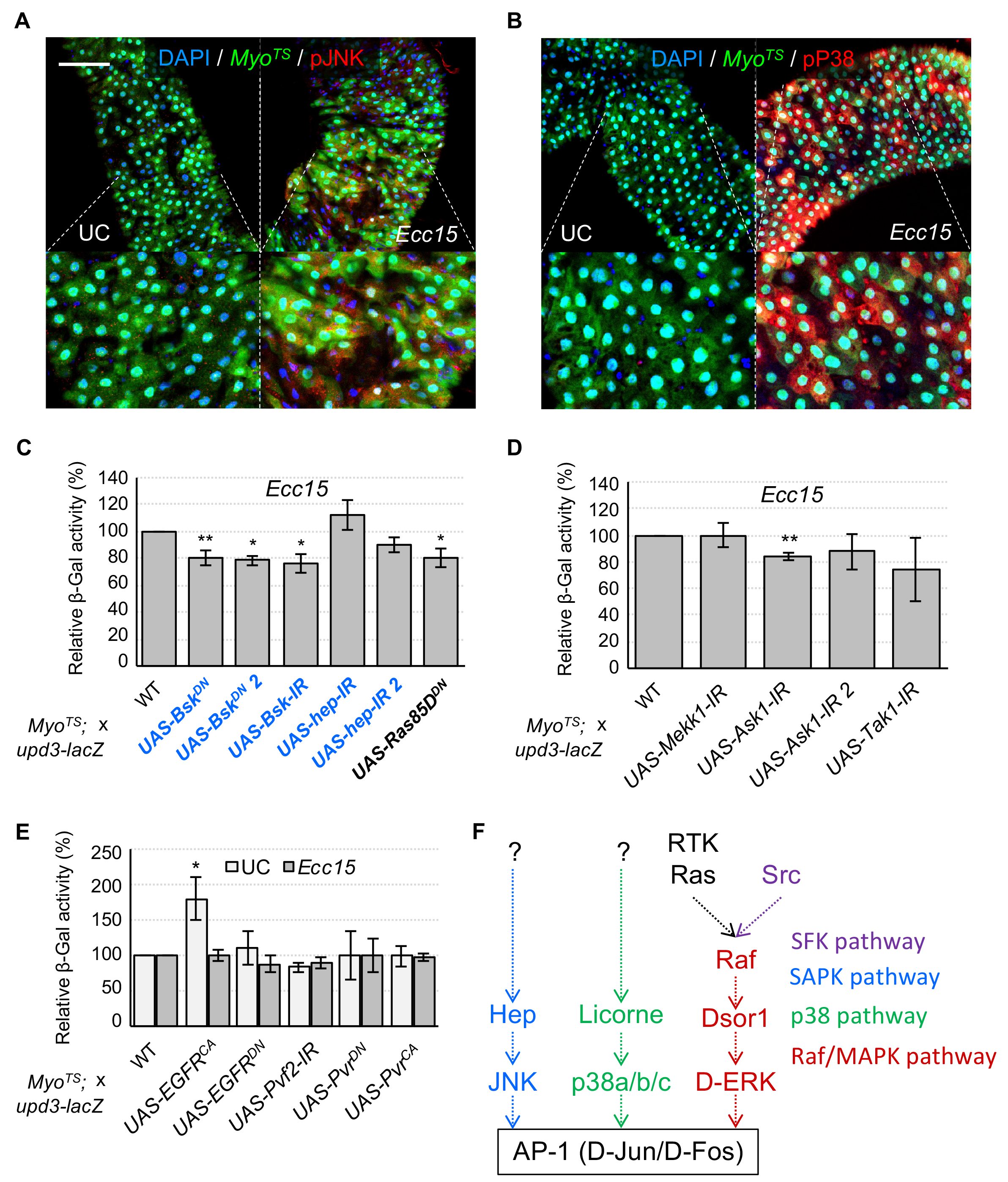

Supplement: S8 Fig — (A, B) Immunostaining against phosphorylated forms of JNKand p38 reveals that these kinases are activated in response to infection in ECs. (C) EC-specific inhibition of Bsk, Hep, or Ras, by RNAi or by expression of dominant negative forms has minimal effect on Ecc15-induced upd3 expression. (D) EC-specific depletion of the MAPKKKs, MEKK1, ASK1, and TAK1 has no major effect on Ecc15-induced upd3 expression. (E) Finally, EC-specific inhibition of EGFR, Pvr, or Pvf2 has no negative effect on upd3-lacZ activity, although activation of EGFR in ECs is sufficient to trigger upd3-lacZ expression. (F) Schematic of the SAPK/MAPK network. The AP-1 complex (D-Jun and D-Fos) is regulated by both Stress Activated Protein Kinase (SAPKs) and Mitogen Activated Protein Kinases (MAPKs). SAPKs lead to the activation of JNK, and MAPKs result in the activation of terminal kinases, including p38 and ERK. Statistical significance: mean values of at least 3 repeats are represented ± SE. *p<0.05, **p<0.01, ***p<0.001 (student’s t test). (TIF) [file pgen.1007091.s008.tif]

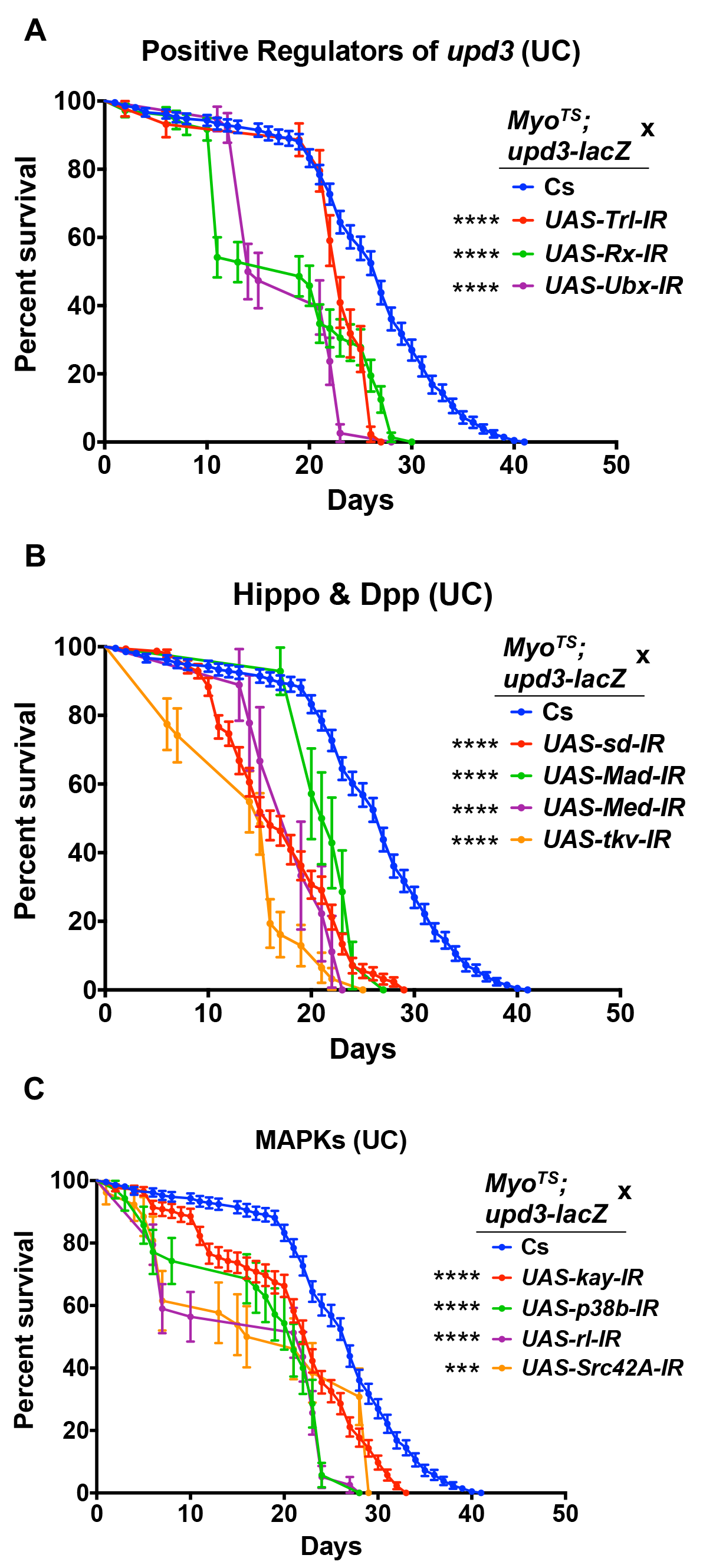

Supplement: S9 Fig — (A-C) RNAi mediated knockdown of epigenetic regulators and homeobox genes (A), Hippo and Dpp pathway genes (B), or SAPK and MAPK constituents (C) reduces the lifespan of unchallenged flies. Curves represent averaged survival ± SE. *p<0.0332, **p<0.0021, ***p<0.0002, ****p<0.0001 (Log-rank test). (TIF) [file pgen.1007091.s009.tif]

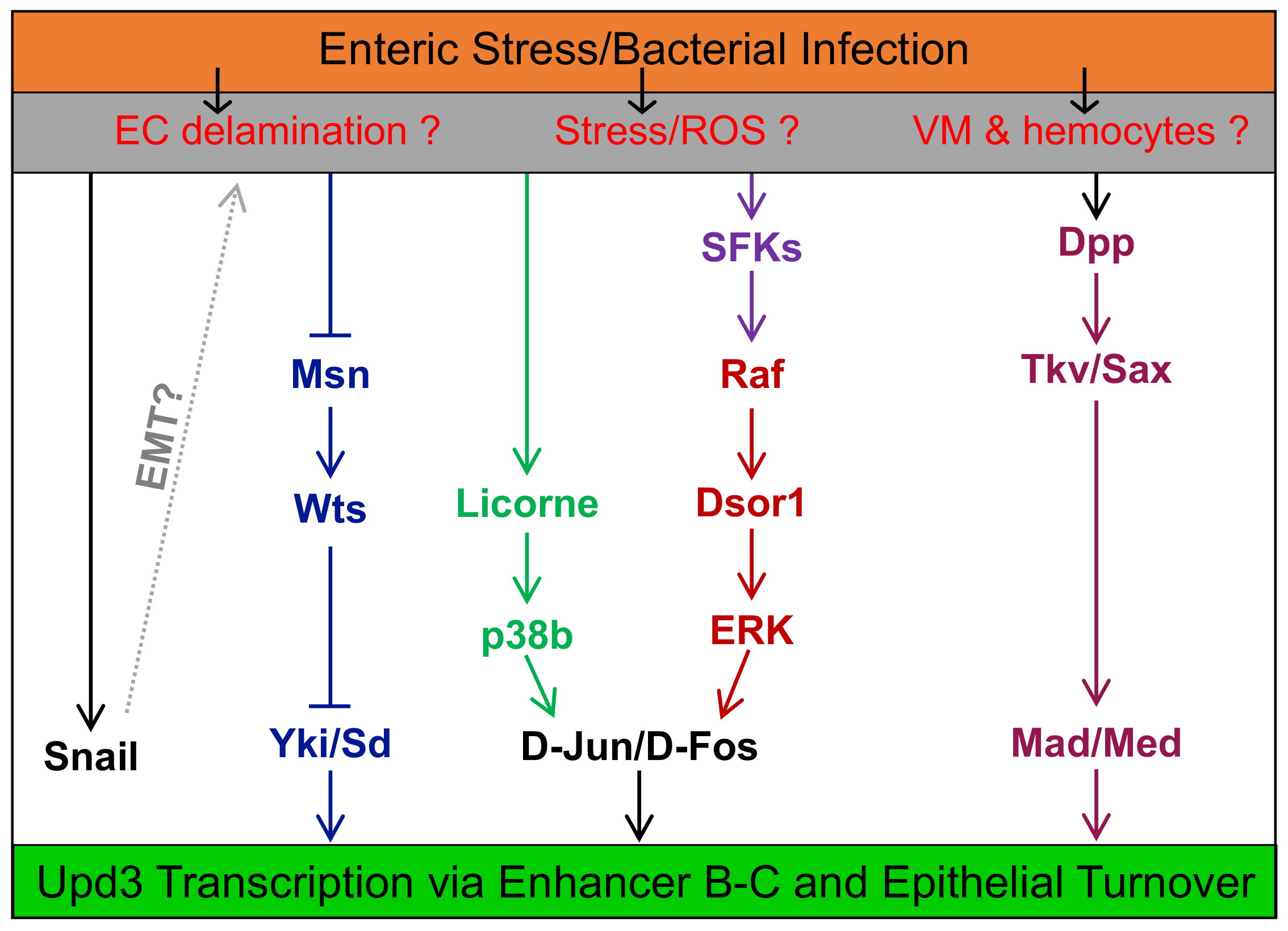

Supplement: S10 Fig — Schematic representation of the pathways that control upd3 transcription in ECs during intestinal trauma. Biotic and abiotic stresses, as well as the responsive ROS production, induce epithelial cell extrusion and cell death. The Sna TF may act as an integral component of cellular extrusion by negatively regulating cellular adhesion. SFK and MAPK pathways are activated by cellular stress, and converge on the activation of D-Fos and D-Jun TFs. The Hippo pathway likely responds to tissue loss in the midgut by removing the inhibition of the Yki and Sd TF complex. (TIF) [file pgen.1007091.s010.tif]
